# Multi-wavelength Raman microscopy of nickel-based electron transport in cable bacteria

**DOI:** 10.3389/fmicb.2024.1208033

**Published:** 2024-03-08

**Authors:** Bent Smets, Henricus T. S. Boschker, Maxwell T. Wetherington, Gérald Lelong, Silvia Hidalgo-Martinez, Lubos Polerecky, Gert Nuyts, Karolien De Wael, Filip J. R. Meysman

**Affiliations:** ^1^Department of Biology, University of Antwerp, Antwerp, Belgium; ^2^Department of Biotechnology, Delft University of Technology, Delft, Netherlands; ^3^Materials Characterization Laboratory, Pennsylvania State University, State College, PA, United States; ^4^Institut de Minéralogie, de Physique des Matériaux et Cosmochimie (IMPMC), Sorbonne Universités, France—Muséum National d’Histoire Naturelle, Paris, France; ^5^Department of Earth Sciences, Utrecht University, Utrecht, Netherlands; ^6^Department of Bioscience Engineering, University of Antwerp, Antwerp, Belgium; ^7^Department of Physics, University of Antwerp, Antwerp, Belgium

**Keywords:** Raman microscopy, cable bacteria, nickel cofactor, metalloprotein, long-distance electron transport

## Abstract

Cable bacteria embed a network of conductive protein fibers in their cell envelope that efficiently guides electron transport over distances spanning up to several centimeters. This form of long-distance electron transport is unique in biology and is mediated by a metalloprotein with a sulfur-coordinated nickel (Ni) cofactor. However, the molecular structure of this cofactor remains presently unknown. Here, we applied multi-wavelength Raman microscopy to identify cell compounds linked to the unique cable bacterium physiology, combined with stable isotope labeling, and orientation-dependent and ultralow-frequency Raman microscopy to gain insight into the structure and organization of this novel Ni-cofactor. Raman spectra of native cable bacterium filaments reveal vibrational modes originating from cytochromes, polyphosphate granules, proteins, as well as the Ni-cofactor. After selective extraction of the conductive fiber network from the cell envelope, the Raman spectrum becomes simpler, and primarily retains vibrational modes associated with the Ni-cofactor. These Ni-cofactor modes exhibit intense Raman scattering as well as a strong orientation-dependent response. The signal intensity is particularly elevated when the polarization of incident laser light is parallel to the direction of the conductive fibers. This orientation dependence allows to selectively identify the modes that are associated with the Ni-cofactor. We identified 13 such modes, some of which display strong Raman signals across the entire range of applied wavelengths (405–1,064 nm). Assignment of vibrational modes, supported by stable isotope labeling, suggest that the structure of the Ni-cofactor shares a resemblance with that of nickel bis(1,2-dithiolene) complexes. Overall, our results indicate that cable bacteria have evolved a unique cofactor structure that does not resemble any of the known Ni-cofactors in biology.

## 1 Introduction

Cable bacteria are filamentous, sulfur-oxidizing bacteria that thrive in marine and freshwater sediments worldwide (Malkin et al., 2014; Risgaard-Petersen et al., 2015). Cable bacteria stand out among the prokaryotes as they have the unique capability to efficiently transport electrons over distances spanning up to several centimeters (Nielsen et al., 2010; Pfeffer et al., 2012; Bjerg et al., 2018; Meysman et al., 2019). This long-distance electron transport (LDET) runs through a network of protein fibers that are embedded in the periplasm (Cornelissen et al., 2018). These fibers run in parallel and continuously along the full length of cable bacterium filaments and act like a network of electrical power lines (Meysman et al., 2019). The fibers display an unprecedented electrical conductivity for a biological material exceeding 100 S/cm and thus present a promising material for the future development of sustainable, lightweight, and biodegradable electronics (Meysman et al., 2019; Boschker et al., 2021).

The nature and composition of the highly conductive protein fibers in cable bacteria is currently poorly understood. Recent analyses indicate that the protein fibers are ~50 nm in diameter (Cornelissen et al., 2018), and consist of a conductive core enveloped by an insulating protein layer (Boschker et al., 2021). The conductive core harbors a metalloprotein with a sulfur-coordinated nickel (Ni) cofactor (Boschker et al., 2021). The presence of Ni is remarkable since biological electron transport through protein structures is thought to be exclusively mediated by copper and iron. All currently known metalloproteins involved in multi-step electron hopping through proteins feature redox centers that include either Fe-containing (e.g., heme groups, Fe-S clusters) or Cu-containing domains (e.g., cupredoxin), but never Ni-containing cofactors (Liu et al., 2014). Still, microorganisms do produce Ni-based metalloproteins, which are considered ancient archaeal and bacterial enzymes that evolved in the Archean when the earth’s atmosphere was devoid of oxygen. However, all currently known Ni metalloproteins serve metabolic functions that are different from electron transport, typically catalyzing reactions involving gases like hydrogen (NiFe hydrogenase), carbon monoxide (CO dehydrogenase), or oxygen (Ni superoxide dismutase; Alfano and Cavazza, 2020; Fontecilla-Camps, 2022). Therefore, it appears cable bacteria have evolved a unique cofactor in order to sustain an exceptional form of long-range electron transport. However, the molecular structure of this cofactor remains unclear.

Raman spectroscopy provides a non-destructive way of obtaining structural information about biomolecules and has been applied extensively to elucidate the structure of metal cofactors (Shiemke et al., 1983; Han et al., 1989; Hu et al., 1993; Garton et al., 1997; Fiedler et al., 2005; Shafaat et al., 2012). In classical Raman spectroscopy, molecules are irradiated with a monochromatic light source and inelastically scattered photons are detected, which are characteristic of molecular vibrations and thus provide a structural fingerprint of molecules (Ferraro et al., 2003; Nakamoto, 2008). Yet, inelastic scattering is a rare event and yields a low signal intensity. Therefore, in resonance Raman spectroscopy, one tunes the wavelength of a laser source to coincide with an electronic transition of a chromophore group in the target molecule. According to the Kramers-Kronig relations, an increase in absorption, described by the real part of the molecule’s complex dielectric function, yields a proportional increase in the imaginary part of the dielectric function, and consequently, the Raman scattering efficiency (Hassing and Sonnich Mortensen, 1980). So, by carefully choosing the wavelength, one can selectively enhance the signals of certain Raman-active modes within the molecule by a factor of up to 10^6^ (Ferraro et al., 2003; Nakamoto, 2008). This way, resonance Raman scattering facilitates the detection of biomolecules at low concentrations in single bacterial cells. Moreover, metal cofactors often exhibit electronic transitions in the visible range of the electromagnetic spectrum, making them prime candidates to study with resonance Raman spectroscopy (Czernuszewicz, 1993).

Here, we employ Raman microscopy with multiple laser wavelengths to analyze the biochemical make-up of cable bacterium cells and to study the broad Raman response of the Ni-cofactor. Previously, it has been shown that cable bacteria produce a unique Raman fingerprint attributed to distinct vibrational modes in the Ni-cofactor (Boschker et al., 2021). However, only two laser wavelengths (532 and 785 nm) were used in these experiments (Bjerg et al., 2018; Boschker et al., 2021). Raman spectroscopy at different incident laser wavelengths enables to differentiate different structural moieties, depending on whether they are or not resonantly enhanced at a given laser wavelength. Therefore, we employed an elaborate multi-wavelength Raman approach to enable an in-depth investigation of the vibrational modes of the novel Ni-cofactor and other cell constituents linked to the intriguing physiology of cable bacteria. We combine this multi-wavelength approach with both ultralow-frequency and orientation-dependent Raman microscopy, as well as stable isotope labeling for a thorough investigation of the molecular structure and spatial arrangement of this Ni-cofactor.

## 2 Materials and methods

### 2.1 Cable bacteria cultivation

Natural marine sediment was collected from the creek bed in a salt marsh (51.4391°N, 4.1697°E; Rattekaai, the Netherlands). Sediment was sieved (1.4 mm stainless steel mesh) to remove debris, plant material, and fauna, homogenized by mixing, and subsequently added into transparent PVC core liner tubes (36 mm diameter, 100 mm height) that were sealed at the bottom with a rubber stopper. These sediment cores were incubated in plastic containers filled with artificial seawater (salinity 30) that was continuously aerated. Incubations were conducted in the dark at a constant temperature of 20°C. Thick cable bacteria (*ca.* 4 μm diameter) developed within 4 weeks and were identified as *Candidatus* Electrothrix gigas based on size and morphology (Geelhoed et al., 2023).

### 2.2 Sample preparation

**Figure 1:**
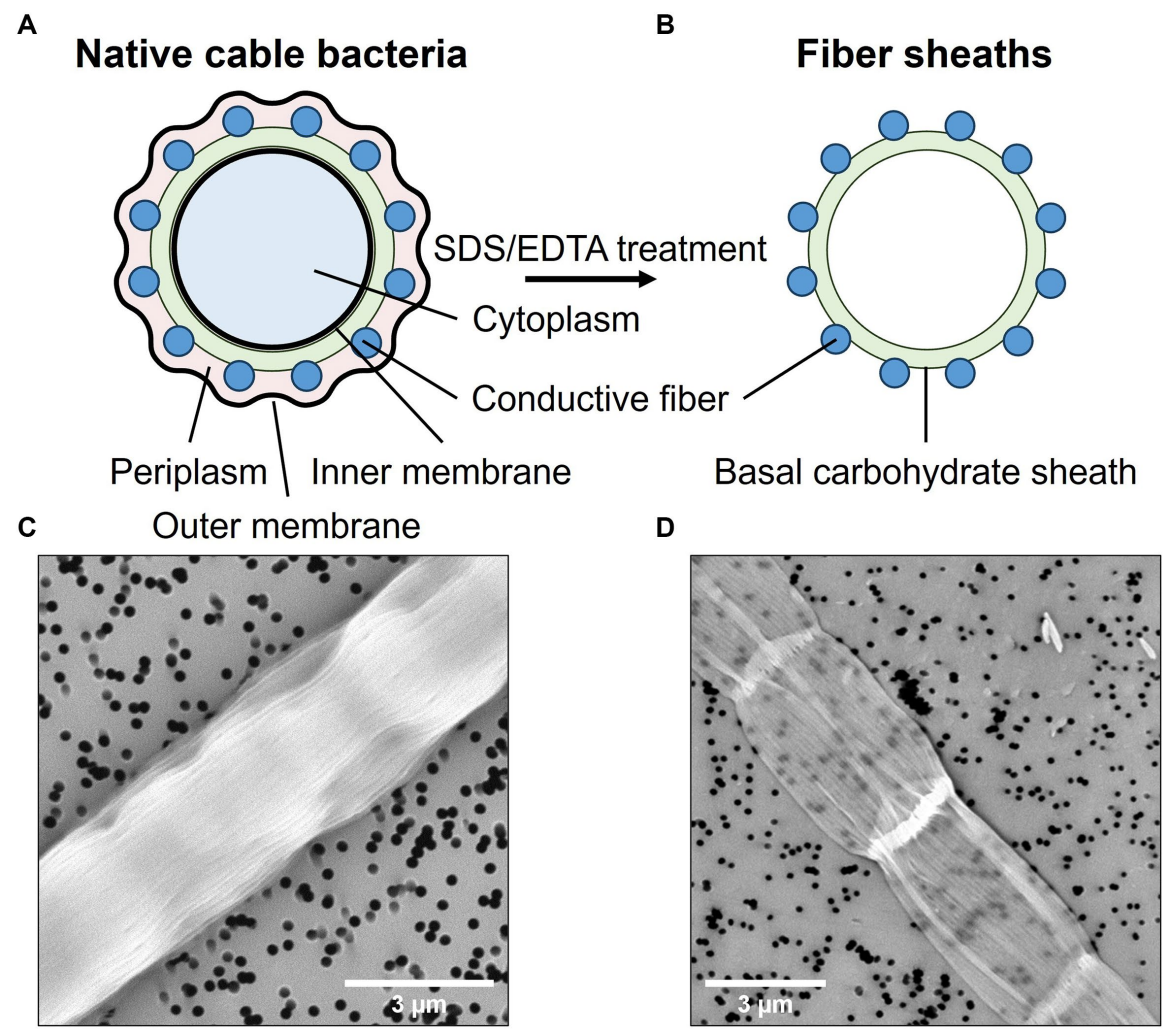
Native cable bacterium filaments were subjected to an extraction procedure that removes the cell content and membranes, leaving only the conductive protein fibers embedded in a thin, basal carbohydrate sheath. Top panels show a schematic representation of a cross-sections across a native filament **(A)** and a fiber skeleton **(B)**. Bottom panels depict SEM micrographs of a native cable bacterium filament **(C)** and a fiber skeleton **(D)**. The black dots are holes in the polycarbonate filter substrate.

Cable bacterium filaments were harvested from the top layer of the sediment and manipulated under a stereomicroscope with custom-made, small glass hooks. Individual filaments were washed to remove sediment particles and salts by transferring them six times between droplets (∼20 μL) of clean MilliQ (mQ) water. The resulting samples are referred to as “native cable bacteria” (Figures 1A,C). In a subsequent step, the conductive fiber network was isolated from native cable bacterium filaments through a chemical extraction procedure. This procedure yields so-called “fiber skeletons” (Figures 1B,D), a cylindrical sheath structure in which the conductive fibers are parallelly embedded in a carbohydrate sheath (Cornelissen et al., 2018). Fiber skeletons remain equally conductive after extraction, indicating that the fiber structure remains unaltered and functional (Meysman et al., 2019). To produce fiber skeletons, native cable bacterium filaments were submerged for 10 min in a droplet (∼20 μL) of 1% (w/w) sodium dodecyl sulfate (SDS) to remove the cell membranes and cytoplasmic cell content. After 10 min, filaments were washed six times in fresh droplets of mQ water to rinse off cell debris and SDS. Next, filaments were transferred to a droplet (∼20 μL) of 1 mM ethylenediaminetetraacetic acid (EDTA) and left for 10 min to remove leftover SDS. Finally, filaments were washed six times in droplets of fresh mQ to obtain clean fiber skeletons. Extraction quality was verified by scanning electron microscopy (SEM). Filaments were fixed onto polycarbonate filters and sputtered (Polaron E5100 sputter coater) for 30 s at a current of 13 mA and pressure of 0.13 mbar. Samples were imaged with a Phenom ProX SEM (Phenom-World, the Netherlands) with a beam energy of 10 keV (Figures 1C,D).

### 2.3 Multi-wavelength and ultralow-frequency Raman microscopy

**Figure 2:**
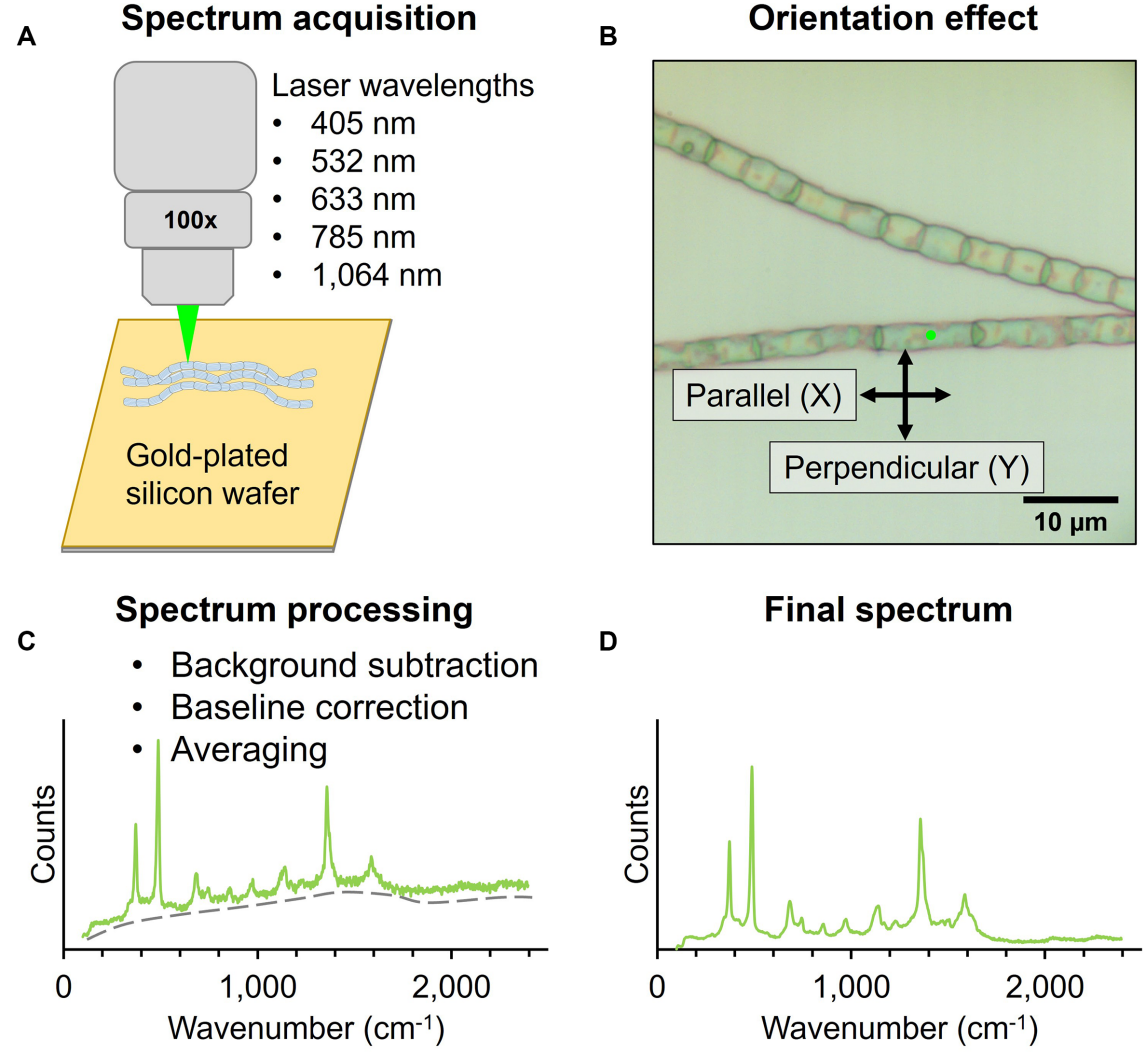
Workflow for Raman microscopy measurements of native cable bacteria and fiber skeletons. **(A)** Filaments were deposited on a gold-plated silicon wafer for Raman spectrum acquisition with five wavelengths. **(B)** The laser polarization (black arrows) was either parallel (X) or perpendicular (Y) to the filament orientation (X). Spectra were collected with the incident laser light (green dot) focused on the central section of cable bacterium cells. **(C)** Acquired spectra were background- and baseline-corrected and averaged to obtain a final spectrum **(D)**.

For Raman microscopy, native cable bacterium filaments and fiber skeletons were deposited on a 7 × 7 mm piece of gold-coated silicon wafer (100 nm gold layer, Platypus Technologies) and air-dried (Figure 2). Raman spectra were collected by focusing the laser spot on the central section of individual cells in cable bacterium filaments. Additionally, the bare gold-plated silicon wafer was measured to determine the background signal.

Two Raman microscope systems were used for data collection: a Renishaw inVia™ Qontor® and a Horiba LabRam HR Evolution Confocal Raman microscope. The Renishaw inVia™ Qontor® microscope was equipped with four Renishaw solid-state lasers: 405 nm laser, 532 nm laser, 785 nm laser, and 1,064 nm laser. A 100x microscopy objective (NA 0.90), and confocal aperture of 65 μm delivered the laser light to the sample and captured Raman scattered photons. Appropriate gratings were installed for each laser to ensure an optimal signal response and spectral resolution of approximately 1 cm^−1^. Raman scattered photons in the visible range were detected with a Renishaw Centrus Charge Coupled Device (CCD) detector, while an Andor Indium Gallium Arsenide photodiode (InGaAs) detector captured photons in the infrared range. Both detectors were thermo-electrically cooled to −70°C. The Horiba LabRam HR Evolution confocal Raman microscope was equipped with a 633 nm laser and 785 nm laser (ThorLabs HRP350-EC). The optical components included a 100x (NA 0.9) objective lens, confocal aperture of 50 μm, and 600 gr/mm grating. Raman scattered light was quantified with a back-illuminated deep-depleted Si array (1,024 × 512 pixel) detector. For ultralow-frequency (ULF) measurements with the 785 nm laser, a dedicated ultralow-frequency filter set was installed to enable the detection of low-energy Raman signals down to 50 cm^−1^. Optimized acquisition parameters for both Raman systems are included in Table 1. All laser sources produced horizontally (X) polarized light by default. All Raman spectra were collected in backscattering conformation, without an analyzer (Z(X,-) $\bar{Z}$ ), unless noted otherwise.

**Table 1:** Optimized Raman spectrum acquisition parameters.

Laser	Grating (gr/mm)	Power (mW)	Acquisition time (s)	Range (cm^−1^)
405 nm	2,400	≈1.5	1	100–3,200
532 nm	1,800	≈2.5	30	100–3,200
633 nm	600	≈5	1	70–2,200
785 nm	1,200	≈25	40	100–3,200
1,064 nm	830	≈200	90	100–3,200

### 2.4 Stable isotope labeling and NanoSIMS

The Ni-cofactor in cable bacteria exhibits several vibrational modes that cannot be annotated with confidence (Boschker et al., 2021). To investigate the molecular origin of these modes, we employed stable isotope probing with ^13^C or ^15^N followed by Raman microscopy to analyze peak shifts caused by the potential incorporation of isotopes in the Ni-cofactor. We then used NanoSIMS analysis to verify and quantify the isotope labeling in the cable bacterium biomass and correlate the level of isotope labeling achieved with the observed shift in the Raman modes. Hereto, we selected sediment cores (36 mm diameter, 100 mm height) with actively growing populations of cable bacteria, as verified by microsensor profiling (Geerlings et al., 2020). Per core, 3 plastic cylinders (10 mm diameter, 50 mm height) were inserted into the sediment to a depth of approximately 45 mm to make “subcores.” Labeled ^13^C-sodium bicarbonate (NaH^13^CO_3_; Cambridge Isotope Laboratories) or ^15^N-ammonium chloride (^15^NH_4_Cl; Eurisotop) were dissolved in sterile, artificial seawater to obtain a 400 mM NaH^13^CO_3_ and 500 mM ^15^NH_4_Cl solution. At the start of the labeling incubation, 500 μL of either the NaH^13^CO_3_ or ^15^NH_4_Cl solution was injected per subcore using a 50 μL gas chromatography syringe, while minimizing sediment disturbance. Labeled cores were stored in an airtight container that was aerated once a day. Sedimentary uptake of unlabeled atmospheric CO_2_ or N_2_ was reduced by adding a tissue soaked in labeled, artificial seawater to the container (this ensuring labeling in the gas phase of the container). After 4 days of incubation, cable bacterium filaments were individually harvested from the sediment for fiber skeleton extraction. Clean fiber skeletons were deposited on a 7 × 7 mm piece of gold-coated silicon wafer (Platypus Technologies) and left to dry overnight. Raman spectra were recorded the next day. After Raman spectrum collection, the degree of ^13^C or ^15^N isotope labeling was determined. For this, the same fiber skeleton positions from which Raman spectra were collected earlier were examined with a NanoSIMS 50 L (Cameca, France) as described in Geerlings et al. (2020). The wafer with samples was placed on a stub and inserted in the vacuum chamber of the NanoSIMS 50 L. In the vacuum chamber, a primary Cs^+^ ion beam was used to repeatedly scan the sample, while analyzing secondary ions ejected from the sample with a high-resolution mass spectrometer. The regions of interest were first visualized with SEM before being pre-sputtered with Cs^+^ ions until secondary ion yields stabilized. Next, multiple scans of 20 × 20 μm were made with the primary Cs^+^ ion beam (beam size: 130 nm, energy: 16 keV, current 0.5–10 pA) and measuring secondary ion counts of ^16^O, ^12^C_2_, ^12^C^13^C, ^12^C^14^N, ^12^C^15^N, ^31^P, and ^32^S. Finally, the ^13^C or ^15^N labeling degree in the fiber skeletons was calculated with the Look@NanoSIMS tool (Polerecky et al., 2012) using the following ratios: 0.5*^12^C^13^C/(^12^C_2_ + 0.5*^12^C^13^C) and ^12^C^15^N/(^12^C^15^N + ^12^C^14^N). These ratios were averaged over the whole scanned area of the filament.

### 2.5 Orientation-dependent Raman microscopy

The orientation-dependent response of the Ni-cofactor’s Raman signal was investigated for both native cable bacteria and fiber skeletons. In the Renishaw system, the microscope stage was rotated, so filaments were oriented in either the parallel (X) or perpendicular (Y) direction to the X-polarized light of the 405, 532, and 785 nm laser sources. The Horiba LabRam HR microscope system utilized an automated 1/2 waveplate in the common path to change the polarization of the 633 nm laser to either parallel or perpendicular to the orientation of filaments in the field of view. Note that the conductive fibers, and hence the electron currents, always run in parallel to the longitudinal axis of the filaments (Meysman et al., 2019).

### 2.6 Processing of Raman spectra

Raman spectrum processing included the removal of cosmic ray spikes, background subtraction and baseline correction. Cosmic ray removal was done in the WiRe™ software (version 5, Renishaw). Background subtraction and baseline correction was done in R (version 4.2.2; R Core Team, 2018). Background spectra were averaged and subtracted from each individual sample spectrum. Baseline correction was done with the R package “baseline” (Liland et al., 2010) using asymmetric least squares baseline correction, which is based on 2nd derivative constrained weighted regression.

### 2.7 UV-Vis-NIR absorption spectroscopy

Native cable bacterium filaments and fiber skeletons were prepared as for Raman microscopy and were either analyzed in dried or wet state. To this end, multiple filaments were deposited together in large, dense clumps on fused quartz microscopy slides (Micro-Tec GE124 fused quartz) and air-dried. Samples were wetted by adding a thin layer of mQ on the microscopy slide after filament deposition, covering the sample with a fused quartz coverslip, and sealing the edges with nail polish. The thin water layer on the microscopy slide reduced the reflectance of the fused quartz substrate.

Absorption spectra were measured with a Lambda 1050 spectrophotometer® (Perkin-Elmer) equipped with a Cassegrain-type microscope (Chassé et al., 2015). Light was focused to a focal spot of 150 μm onto dense spots with either cable bacteria or fiber skeletons, and spots without material for background correction. Transmitted light was collected and quantified with an array of three detectors: a CCD, an InGaAs photodiode, and a polycrystalline lead sulfide (PbS) detector. These detectors covered a spectral range from 210 to 2,500 nm. Spectrum processing was limited to background subtraction and averaging of spectra recorded under identical conditions.

## 3 Results

### 3.1 Multi-wavelength Raman microscopy of native cable bacterium filaments

**Figure 3:**
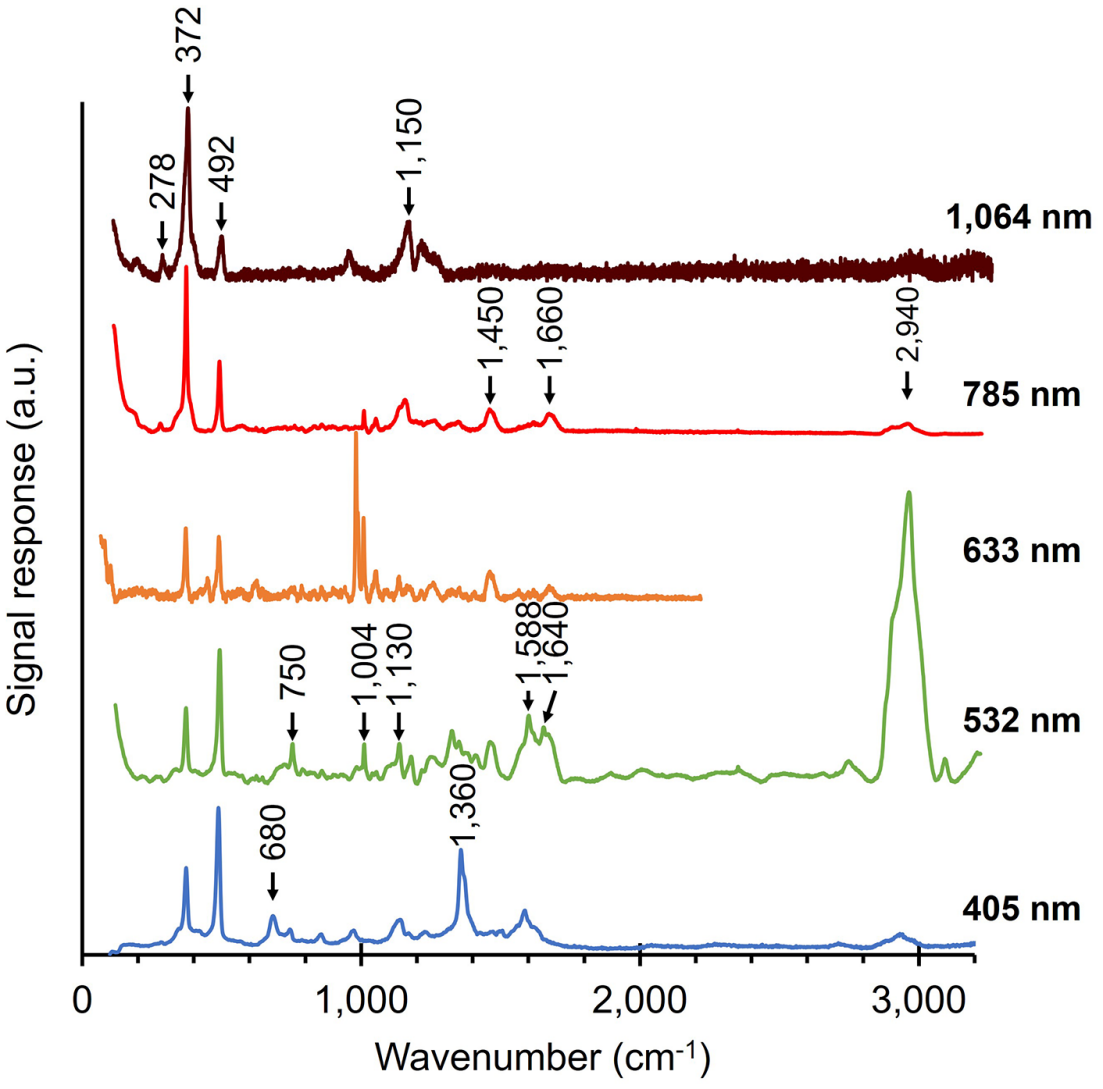
Raman spectra of native cable bacteria were collected with five different wavelengths (indicated on the right). Arrows and numbers indicate relevant modes specific to the fingerprint of cable bacteria. The depicted spectra represent averages of multiple, individually collected spectra. The number of spectra collected per wavelength was: 55 (405 nm), 110 (532 nm), 5 (633 nm), 38 (785 nm), and 70 (1,064 nm). a.u., arbitrary units. Spectra are off-set for clarity.

Native cable bacteria were subjected to Raman microscopy using five different laser wavelengths (Figure 3; Table 1): blue (405 nm), green (532 nm), orange (633 nm), red (785 nm) and infrared (IR; 1,064 nm). For each laser wavelength, individual spectra were collected in many different cells, before being processed and averaged to derive the final spectrum. The resulting spectra show a set of peaks, which can be attributed to vibrational modes that originate from distinct classes of cellular compounds: general protein signals, cytochrome peaks, and modes that uniquely link to the novel Ni-cofactor (peak annotations are provided in Table 2).

**Table 2:** Overview of Raman active modes found in cable bacteria.

Peak	CB	FS	Intensity	Annotation	Ref.
53 cm^−1^	?		Low	Ni-cofactor: annotation uncertain	[1]
95 cm^−1^	?		Low	Ni-cofactor: annotation uncertain	[1]
121 cm^−1^	?		Low	Ni-cofactor: annotation uncertain	[1]
182 cm^−1^			Low	Ni-cofactor: δ(SNiS) − S-Ni-S deformation	[2]
278 cm^−1^			Low	Ni-cofactor: ν(Ni-S)_asym_ − Ni-S stretching	[2]
308 cm^−1^			Low	Ni-cofactor: annotation uncertain	[1]
367 cm^−1^			High	Ni-cofactor: ν(Ni-S)_sym_ − Ni-S stretching	[2, 3]
448 cm^−1^			Low	Ni-cofactor: annotation uncertain	[1]
496 cm^−1^			High	Ni-cofactor: ν(C-S) + ring deformation	[2, 3]
622 cm^−1^			Low	Ni-cofactor: annotation uncertain	[1]
680 cm^−1^			Medium	Cytochromes: ν(C_a_-S)	[4, 5]
750 cm^−1^			Medium	Cytochromes: ν_15_ − Pyrrole breathing	[4, 5]
856 cm^−1^			Low	Tyrosine: Fermi resonance − ν_1_ + 2ν_16a_	[6]
976 cm^−1^			High	Phosphate: ν(PO_4_^3−^) − Symmetric phosphate stretching	[7, 8]
1,004 cm^−1^			Medium	Phenyl alanine: Ring breathing	[9]
1,130 cm^−1^			Medium	Cytochromes: ν_22_ − vibrations of side radicals C_b_-CH_3_	[4, 5]
1,146 cm^−1^			High	Ni-cofactor: ν(C=S) − thiocarbonyl radical stretching	[10]
1,181 cm^−1^			High	Ni-cofactor: ν(C=S) − thiocarbonyl radical stretching	[10]
1,219 cm^−1^			High	Ni-cofactor: ν(C=S) − thiocarbonyl radical stretching	[10]
1,360 cm^−1^			High	Cytochromes: ν_4_ − symmetric pyrrole half-ring stretching	[4, 5]
1,450 cm^−1^			Medium	Carbohydrates, lipids, protein: ρ(CH_2_) − CH_2_ scissoring	[11–14]
1,588 cm^−1^			Medium	Cytochromes: ν_19_ − ν(C_α_C_m_)_asym_	[4, 5]
1,640 cm^−1^			Medium	Cytochromes: ν_10_ − ν(C_α_C_m_)_asym_	[4, 5]
1,660 cm^−1^			Medium	Proteins: ν(C=O) − Carbonyl stretching (Amide I)	[15]
2,940 cm^−1^			High	Carbohydrates, DNA, lipids, proteins: ν(C-H) − C-H stretching	[10, 13, 16, 17]

Dark gray shaded areas indicate modes observed in native cable bacterium filaments (CB) and fiber skeletons (FS). Light gray shaded areas indicate modes attributed to the Ni-cofactor. [1] this work, [2] Johnson (2004), [3] Boschker et al. (2021), [4] Virdis et al. (2014), [5] Milazzo et al. (2018), [6] Siamwiza et al. (1975), [7] Frost et al. (2016), [8] Nkebiwe et al. (2022), [9] Freire et al. (2017), [10] Petrenko et al. (2006), [11] Czamara et al. (2015), [12] Ferraro et al. (2003), [13] Salmaso et al. (1994), [14] Wiercigroch et al. (2017), [15] Miura and Thomas (1995), [16] Kengne-Momo et al. (2012), [17] Prescott et al. (1984). ? indicates vibrational modes which were not examined.

Genome analysis reveals that several cytochrome genes are present in the genome of cable bacteria (Kjeldsen et al., 2019). Cytochromes are known to exhibit resonant Raman scattering as a result of absorption from the Soret (410 nm) and Q (530 nm) bands (Hu et al., 1993; Virdis et al., 2014), which are close to the wavelengths of the blue (405 nm) and green (532 nm) lasers employed. When using these lasers, we indeed detected strong peaks at the expected positions for cytochromes: 680, 750, 1,130, 1,360, 1,588, and 1,640 cm^−1^ (Virdis et al., 2014; Bjerg et al., 2018; Milazzo et al., 2018). In contrast, cytochrome signals were absent in Raman spectra recorded at longer wavelengths (633, 785, and 1,064 nm), consistent with the absence of cytochrome absorption bands beyond 550 nm (Liu and Webster, 1974).

The Raman spectra of native cable bacteria also show signals attributable to generic microbial biomass components. Two characteristic protein modes were identified upon irradiation with the 532, 633, and 785 nm laser. These vibrational modes include the phenyl alanine ring breathing (1,004 cm^−1^) and the Amide I mode (1,660 cm^−1^; Miura and Thomas, 1995; Freire et al., 2017). We observed weaker protein signals in the blue (405 nm) spectrum at 856 and 1,253 cm^−1^, which originate from tyrosine side chains and the Amide III mode of the protein backbone, respectively (Siamwiza et al., 1975; Miura and Thomas, 1995). Other vibrational modes linked to generic, organic compounds were the CH_2_ scissoring mode at 1,450 cm^−1^ detected with the green (532 nm) and red (785 nm) laser, and the C-H stretching modes around 2,940 cm^−1^ present in almost all spectra. These two vibrational modes are present in many cellular constituents like proteins, carbohydrates, DNA, and lipids (Prescott et al., 1984; Salmaso et al., 1994; Ferraro et al., 2003; Kengne-Momo et al., 2012; Czamara et al., 2015; Wiercigroch et al., 2017). Occasionally, an intense peak was observed at 971 cm^−1^, as seen in the orange (633 nm) spectrum. This peak likely originates from phosphate stretching (Frost et al., 2016; Nkebiwe et al., 2022) in the polyphosphate granules that are widely present in cable bacteria (Sulu-Gambari et al., 2016a; Geerlings et al., 2022). However, such polyphosphate granules are not present in every cell (Geerlings et al., 2022), explaining the occasional presence of the 971 cm^−1^ peak in the spectra. A complete annotation list of the Raman spectrum of native cable bacteria is provided in Supplementary Table 1.

A striking feature in the Raman spectra of native cable bacteria are the two peaks at 372 cm^−1^ and 492 cm^−1^ (Figure 3). Boschker et al. (2021) previously observed these peaks and assigned them to vibrational modes of the Ni-cofactor in the conductive fibers. Notably, these two peaks occur prominently in the Raman spectra of all the examined laser wavelengths. The height of these Ni-cofactor peaks with respect to other cell compounds in the spectra suggests the Raman signals are resonantly enhanced across a wide frequency range (Figure 3).

### 3.2 Multi-wavelength Raman microscopy of fiber skeletons

**Figure 4:**
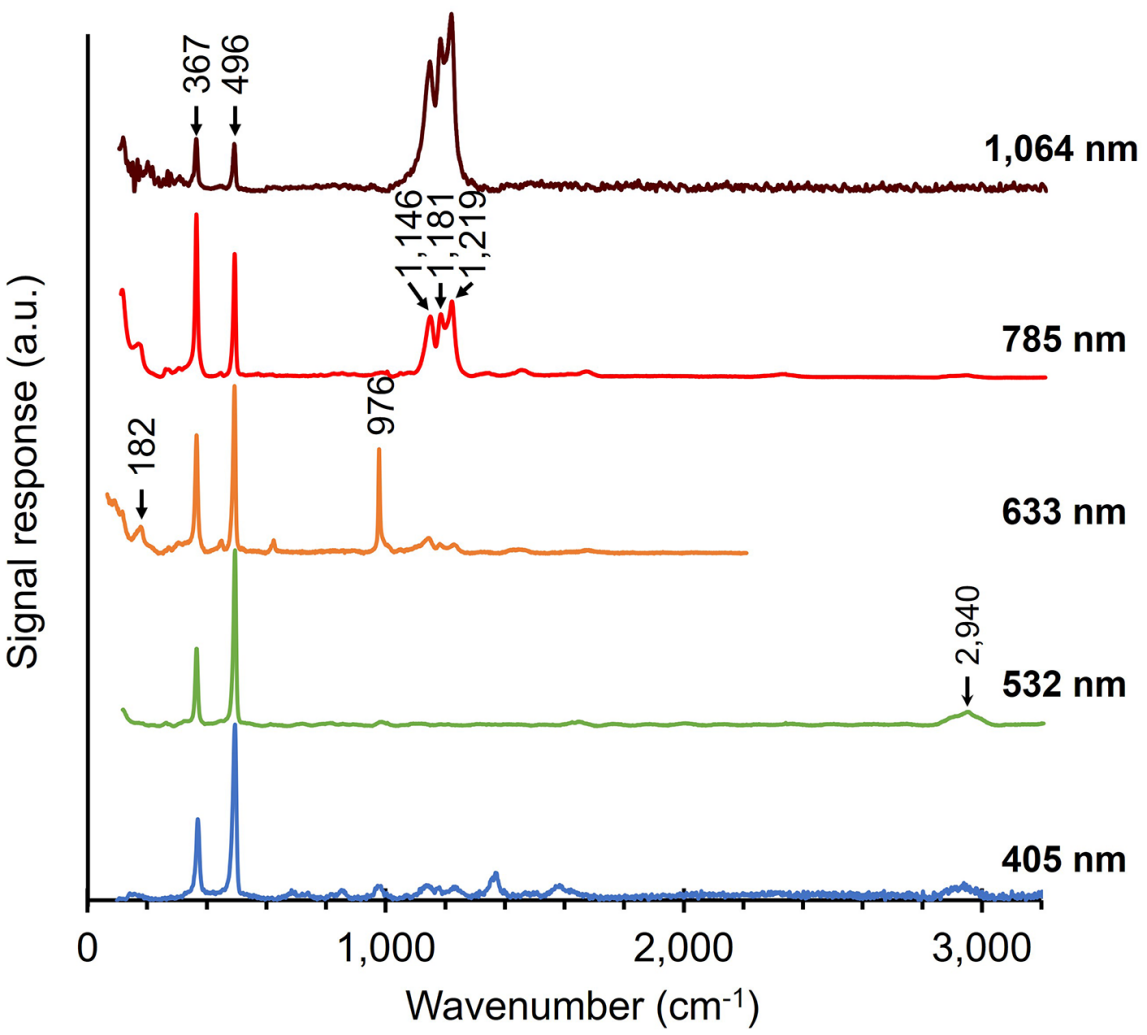
Raman spectra of fiber skeletons isolated from native cable bacteria. Multiple spectra, originating from different spots along fiber skeletons, were acquired for each of the five wavelengths. Arrows and numbers indicate prominent modes in the fiber skeleton samples. The number of collected spectra per wavelength: 50 (405 nm), 52 (532 nm), 5 (633 nm), 60 (785 nm), 150 (1,064 nm). a.u., arbitrary units. Spectra are off-set for clarity.

Raman spectra of fiber skeletons were obtained using the same workflow as that of native cable bacteria. The removal of cellular material during the extraction procedure is clearly reflected in all recorded spectra of fiber skeletons (Figure 4). Overall, fiber skeletons produced a much “simpler” Raman spectrum compared to native cable bacteria. We observed a substantial decrease in the number and magnitude of peaks associated with cytochromes and generic biomass components, though not for the two characteristic modes linked to the Ni-cofactor (i.e., 367 and 496 cm^−1^).

The cytochrome signals disappeared completely in the green (532 nm) spectrum and only small remnant peaks were observed in the blue (405 nm) spectrum. This confirms earlier findings that cytochromes are almost completely removed during the fiber extraction procedure (Meysman et al., 2019). The intensity of modes associated with general cellular compounds also decreased greatly upon extraction. As expected, the removal of the cytoplasm and membranes resulted in a substantial drop in the intensity of the protein-related modes (Phe ring breathing—1,004 cm^−1^ and Amide I—1,660 cm^−1^) and the CH_2_ scissoring (1,450 cm^−1^) and C-H stretching mode (2,940 cm^−1^) in spectra recorded with the green (532 nm), orange (633 nm), and red (785 nm) lasers. Curiously, we also detected the presence of leftover polyphosphate granules in fiber skeletons, as indicated by the strong signal at 976 cm^−1^ in the orange (633 nm) spectrum (Frost et al., 2016; Nkebiwe et al., 2022). This aligns with previous observations that polyphosphate granules are not completely removed during the fiber skeleton extraction procedure (Cornelissen et al., 2018). An annotation list of vibrational modes in both native cable bacteria and fiber skeletons is presented in Table 2.

Upon fiber skeleton extraction, the network of conductive fibers is retained, increasing the relative concentration of the embedded Ni-cofactors. This is directly reflected in the Raman spectra. The two low-frequency peaks in the spectra of native cable bacteria (Figure 3) also dominate all spectra of fiber skeletons (Figure 4). This indicates that the Ni-cofactor, although present at low concentration (Boschker et al., 2021), produces strong, potentially resonant Raman signals across a broad range of wavelengths. Curiously, we observed a peak shift beyond the spectral resolution for both modes in fiber skeletons compared to native cable bacteria. The peak at 372 cm^−1^ shifted down −5 cm^−1^ (372 → 367 cm^−1^), while the 492 cm^−1^ peak shifted up +4 cm^−1^ (492 → 496 cm^−1^) after the extraction procedure. This suggests a (moderate) impact of the extraction on the bond lengths of cofactor, while not affecting its functionality, as fiber skeletons remain equally conductive compared to native cable bacterium filaments (Meysman et al., 2019).

In the Raman spectra of fiber skeletons (Figure 4), three closely spaced peaks become visible in the mid-frequency range (1,146, 1,181, and 1,219 cm^−1^) of the orange (633 nm) spectrum and are very prominent in the red (785 nm) and infrared (1,064 nm) spectrum (Figure 4). These peaks comprise a feature that we refer to as the “castle,” which is absent upon irradiation with the blue (405 nm) and green (532 nm) lasers but increases notably in intensity toward longer wavelengths. Effectively, the castle feature entirely dominates the infrared (1,064 nm) spectrum (Figure 4).

### 3.3 Impact of stable isotope labeling on Raman spectra

**Figure 5:**
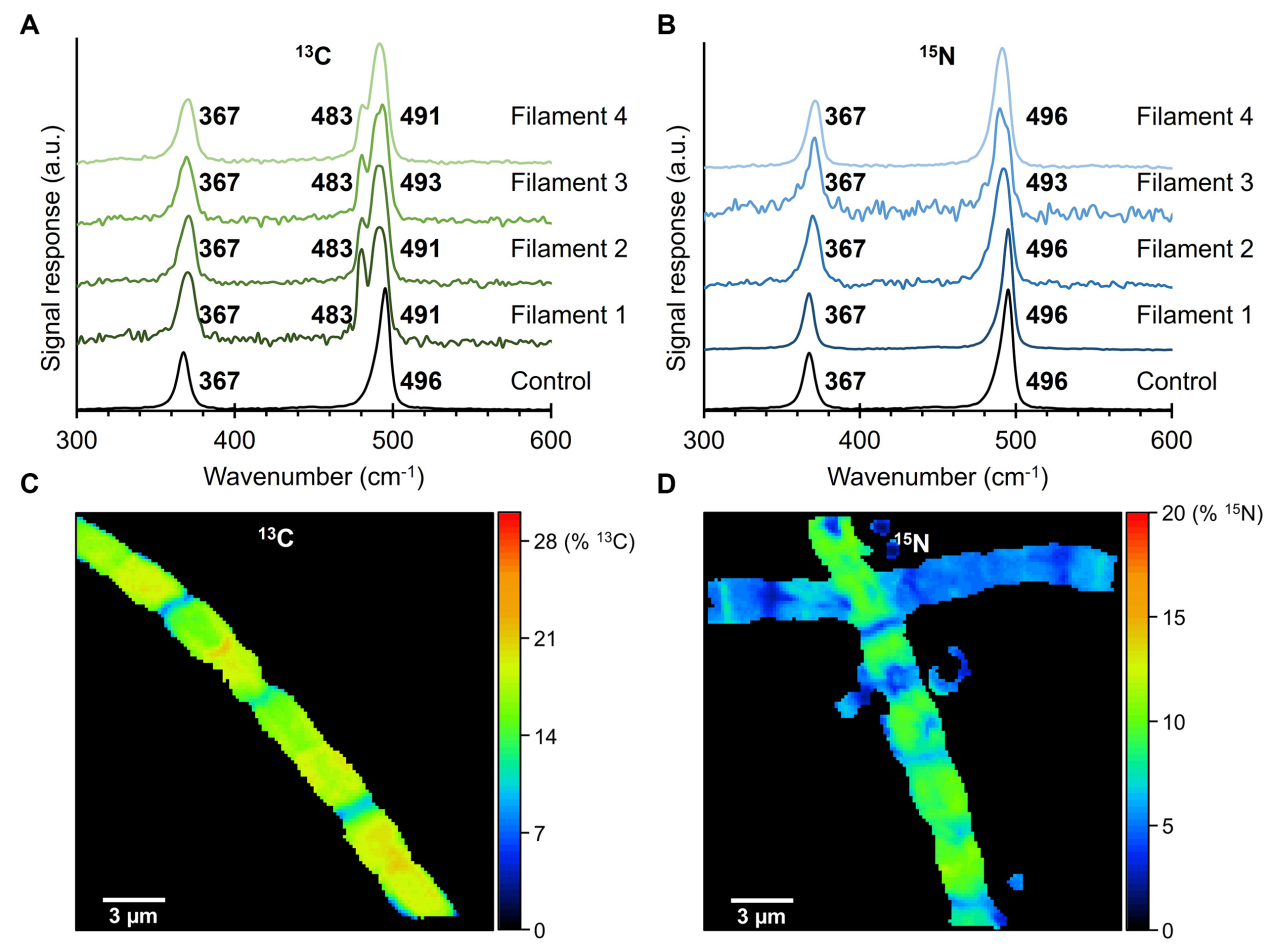
Raman spectra of fiber skeletons isolated from ^13^C- and ^15^N-labeled fiber skeletons. **(A)** Isotopic labeling with ^13^C (NaH^13^CO_3_) resulted in a large (−13 cm^−**1**^) and small (−5 cm^−**1**^) downward shift in the 496 cm^−**1**^ peak of the Ni-cofactor. **(B)** Incorporation of ^15^N in the cable bacterium biomass did not produce peak shifts. Control spectra of non-labeled fiber sheaths are indicated in black. Raman spectra were recorded with a 532 nm laser source. **(C)** The ^13^C-labeling and **(D)** ^15^N-labeling degree in the analyzed fiber skeletons were determined with NanoSIMS.

Fiber skeletons were extracted from cable bacteria that were exposed to isotope labeling with either ^13^C (NaH^13^CO_3_) or ^15^N (^15^NH_4_Cl). Per isotopic label, 4 fiber skeletons were prepared, of which several cells were measured with the 532 nm laser source to determine an average Raman spectrum for each filament (Figure 5). Fiber skeletons obtained from non-labeled sediment incubations were used to collect control Raman spectra.

Comparison of the two dominant low-frequency modes of the Ni-cofactor in the Raman spectra of ^13^C-labeled and control filaments revealed that ^13^C incorporation produced downward shifts of the 496 cm^−1^ peak toward 483 cm^−1^ (−13 cm^−1^) and 491 cm^−1^ (−5 cm^−1^; Figure 5A). These shifts indicate that ^13^C was integrated into the biomass and Ni-cofactor, changing the frequency of the molecular vibration linked to the 496 cm^−1^ peak. Previous ^34^S-labeling experiments also resulted in a downward shift of this mode (Boschker et al., 2021). Consequently, both C and S must be involved in the molecular bond that produces the 496 cm^−1^ mode. The ^13^C labeling did not affect the 367 cm^−1^ peak. Similarly, the Raman spectra of ^15^N-labeled samples were compared to control spectra. The peak positions of the two low-frequency modes of the Ni-cofactor were determined to be at 367 and 496 cm^−1^ in both ^15^N-labeled and control samples (Figure 5B). Therefore, ^15^N incorporation does either not significantly affect the molecular vibrations in the Ni-cofactor, or alternatively, ^15^N-labeling was not successful.

To rule out the second option, samples were analyzed with NanoSIMS to verify and quantify ^13^C or ^15^N incorporation into the cable bacterium biomass. The labeling degree of ^13^C in fiber skeletons was consistent between cells of the same filament (Figure 5C) but varied between individual fiber skeletons. We detected labeling degrees ranging from 7% to 20% ^13^C, while the natural abundance of ^13^C was 1.11%. ^15^N-labeling varied between cells and fiber skeletons (Figure 5D). Still, cable bacteria incorporated ^15^N in their biomass, reaching labeling degrees varying between 1% and 19% ^15^N, while the natural abundance of ^15^N amounted to 0.38%. Thus, NanoSIMS confirms that cable bacteria successfully incorporated both the ^13^C and ^15^N label into their biomass, at labeling degrees sufficient to induce shifts in Raman peaks. Yet, only ^13^C causes a distinct shift in the vibrational modes of the Ni-cofactor.

### 3.4 Ni-cofactor modes display an orientation-dependent response

During Raman spectrum collection, we noticed that the intensity of the five most prominent Ni-cofactor peaks (367, 496, 1,146, 1,181, and 1,219 cm^−1^) varied strongly between filaments. Depending on the orientation and positioning of the filaments on the substrate, the signals associated with the Ni-cofactor changed substantially, while the signals of cytochromes and protein-related modes (Phe ring breathing, Amide I) remained invariant. The conductive fibers run in parallel to the longitudinal axis of the filaments (Figures 1C,D), thus suggesting that the Raman scattering intensity strongly correlates with the orientation of the fibers. Both Raman systems employed in this work (Renishaw inVia™ Qontor® and Horiba LabRam HR Evolution) use laser sources that produce polarized light. To examine this orientation effect systematically, cable bacterium filaments and fiber skeletons were oriented in two different directions: parallel and perpendicular to the polarization of the blue (405 nm), green (532 nm), orange (633 nm), and red (785 nm) laser.

**Figure 6:**
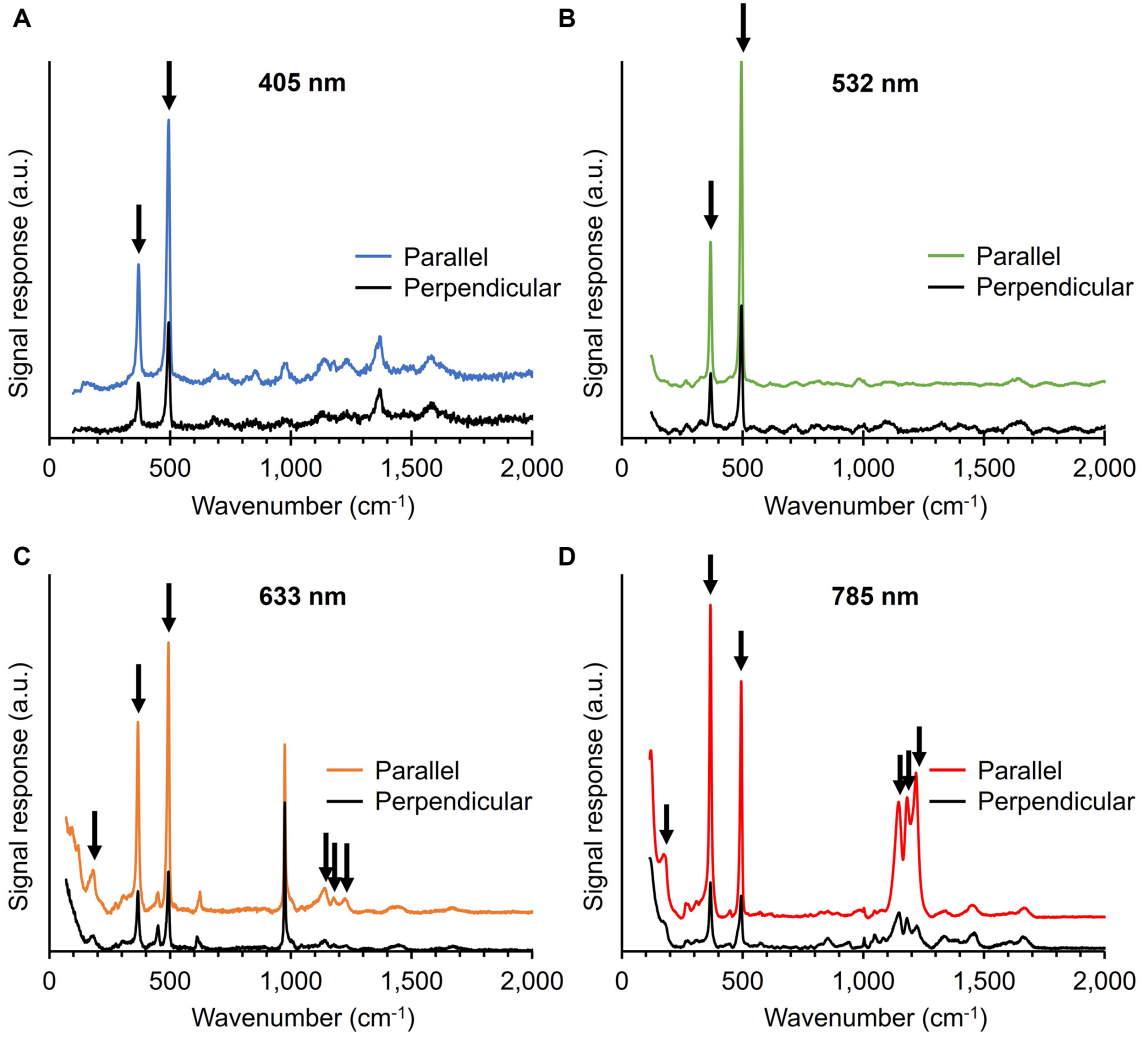
Raman spectra of fiber skeletons recorded with the 405 **(A)**, 532 **(B)**, 633 **(C)**, and 785 nm laser **(D)**. The incident laser light was either polarized parallel or perpendicular to the filament orientation. Vibrational modes associated with the Ni-cofactor, indicated with black arrows, display a strong, orientation-dependent Raman response. Spectra are off-set for clarity.

The five most intense Ni-cofactor modes in fiber skeletons exhibited a two- to six-fold increase in Raman signal intensity when the filament direction was parallel to the laser polarization as compared to the perpendicular conformation (Figure 6; Supplementary Table 2). The magnitude of the orientation-dependent signal response of the Ni-cofactor varied for different modes (Figure 6; Supplementary Figure 1). We determined the sensitivity toward the laser source polarization of the Ni-cofactor modes in fiber skeletons and compared them to the Amide I mode, which should be unaffected by changes in polarization (Supplementary Table 2). This was done by calculating the ratio of the signal intensity of the peak recorded in the parallel conformation (Peak_Parallel_) compared to the intensity of the same peak in perpendicular conformation (Peak_Perpendicular_). An overview of the ratios can be found in Supplementary Table 2. The observed variation in strength of the orientation effect is likely caused by imperfect polarization of the incident laser light and slight deviations in the orientation of the filaments.

The ratio for both low-frequency modes (367 and 496 cm^−1^) was similar at all wavelengths and increased from 2 to 4 when moving toward longer wavelengths. The castle features (1,146, 1,181, and 1,219 cm^−1^) also displayed a clear orientation-dependent response, which was the largest with the red (785 nm) laser. The peaks are 3 to 6 times more intense upon parallel irradiation compared to perpendicular. The sensitivity toward the polarization of the incident light is thus of the same order of magnitude for the five most intense Ni-cofactor-related modes. The Amide I mode (1,660 cm^−1^) displayed a 10% decrease in Raman signal intensity when comparing parallel (X) to perpendicular (Y) irradiation, resulting in a ratio of 0.9. A ratio around 1 is expected since the protein backbone is assumed to have a random orientation in the protein structure of the conductive fibers.

The fact that all these modes consistently display the same, strong orientation dependence indicates that they are linked to the same molecular structure, i.e., the Ni-cofactor. This hypothesis is also supported by orientation-dependent measurements on native cable bacterium filaments. Also here, the signature Ni-cofactor modes (367 and 496 cm^−1^) exhibited a strong change in Raman intensity upon changing the filament orientation. In contrast, other compounds like cytochromes (680, 750, 1,130, 1,360, 1,588, and 1,640 cm^−1^) and proteins (Phe breathing mode—1,004 cm^−1^, Amide I—1,660 cm^−1^), produced Raman signals with a similar intensity upon both parallel and perpendicular irradiation (Supplementary Figure 1). This indicates that orientation-dependent Raman scattering in cable bacteria is unique to the Ni-cofactor in the conductive fibers.

### 3.5 Ultralow-frequency Raman microscopy of fiber skeletons

**Figure 7:**
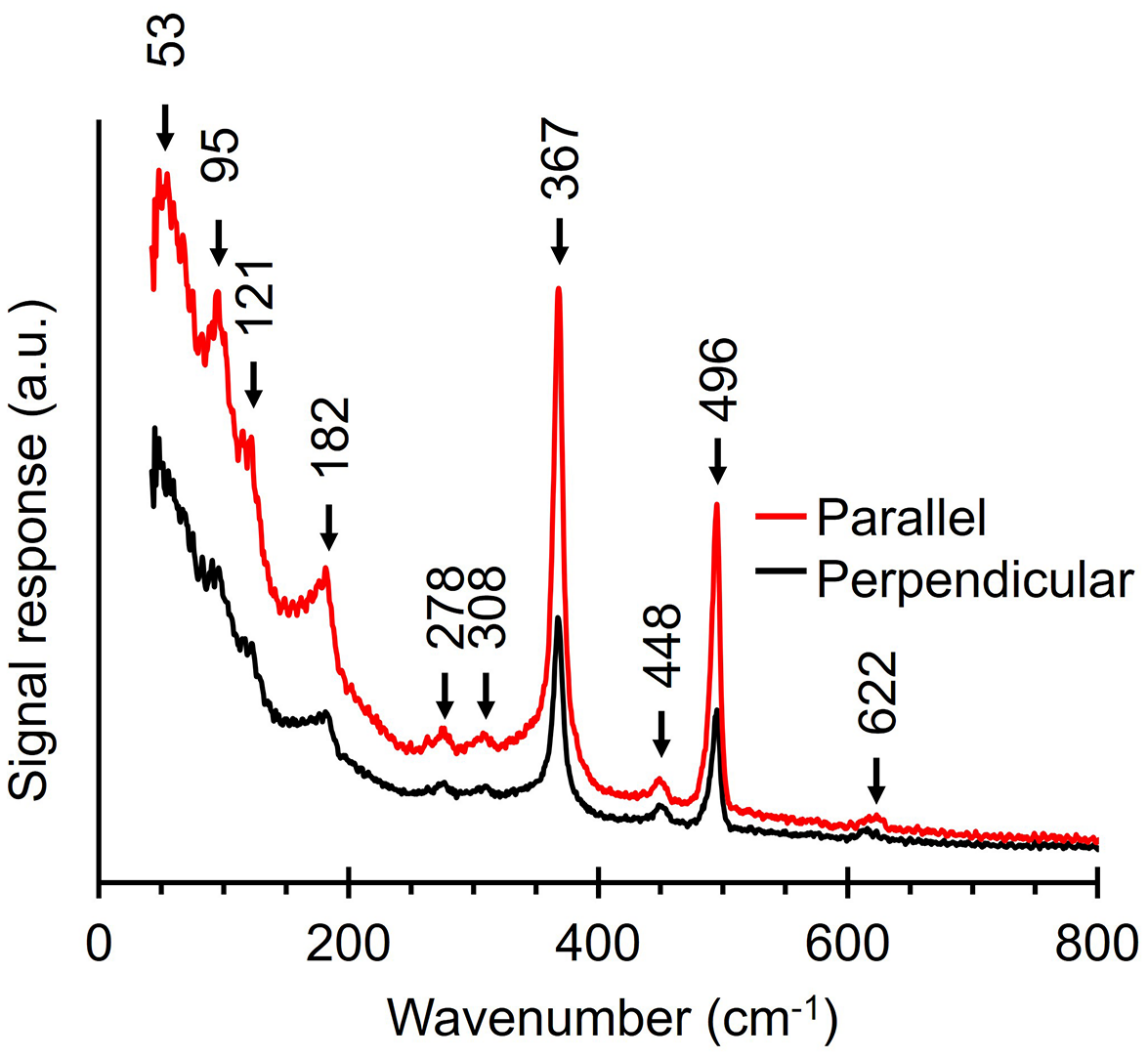
Ultralow-frequency Raman spectra obtained from fiber skeletons with a 785 nm laser source. Red line, laser polarization is parallel to the longitudinal axis of the filament. Black line, laser polarization is perpendicular to the longitudinal axis of the filament.

Upon inspection of the low-frequency region (100–250 cm^−1^), we noticed that this part of the fiber skeleton Raman spectrum featured several weak vibrational modes (Figures 3, 4). These modes are difficult to resolve since they are close to the cut-off frequency of the filters that remove the incident laser light (100 cm^−1^). Hence, we employed ultralow-frequency (ULF) Raman microscopy using the red (785 nm) laser source and an ULF filter set to access low-energy Raman signals down to 50 cm^−1^. To investigate the orientation-dependent Raman response, we tested irradiation of fiber skeletons with both parallel and perpendicularly polarized light. Doing so, we detected several low-frequency vibrational modes that can potentially be linked to the Ni-cofactor (Figure 7). We observed peaks at 53, 95, 121, 182, 278, 308, 448, and 622 cm^−1^ that have not previously been described. The peaks at 95, 121, 182, and 278 cm^−1^ exhibited a clear, orientation-dependent behavior as they were more intense upon parallel irradiation. As a result, we assign these peaks to molecular vibrations in the Ni-cofactor. The other peaks are smaller, making the orientation-dependent response less clear. Nevertheless, due to the relative enrichment of Ni-cofactors in fiber skeletons, it is likely these modes also originate from the cofactor. So far, we can only annotate two of these newly discovered modes with confidence, as they are also observed in certain Ni coordination complexes (see discussion below): S-Ni-S deformation (182 cm^−1^) and asymmetric Ni-S stretching (278 cm^−1^; Johnson, 2004).

### 3.6 UV-Vis-NIR absorption spectroscopy

The strong Raman signals produced by the Ni-cofactor could result from resonant Raman scattering across the range of applied wavelengths (405–1,064 nm). Since resonant scattering occurs when the energy of incident photons is close to an electronic transition in the molecule, the Ni-cofactor must enable transitions and/or charge-transfers in this spectral range. The Raman scattering intensity is particularly strong in the (near)-infrared (NIR) region (785–1,064 nm), as indicated by the three “castle” modes that prominently emerge (Figure 4). As a result, we expect strong absorption in the NIR region caused by the Ni-cofactor. To uncover these potential transitions and charge-transfers causing absorption, we subjected native cable bacteria and fiber skeletons to UV-Vis-NIR absorption spectroscopy.

**Figure 8:**
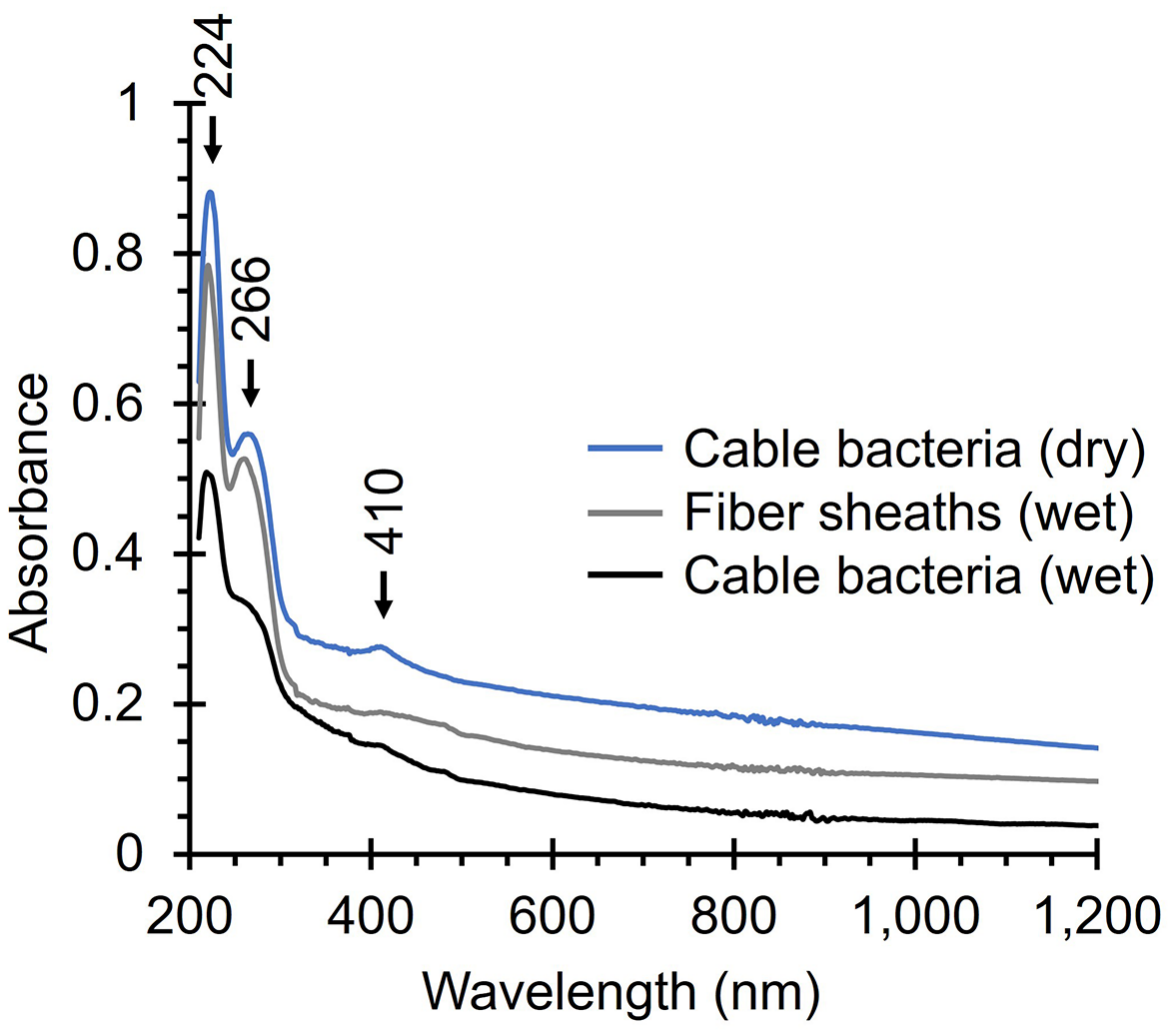
UV-Vis-NIR absorption spectra of native cable bacteria and fiber skeletons. Relevant absorption peaks are indicated with numbers and arrows. Three spectra were recorded per condition, background-corrected, and averaged. The noise between 800 and 900 nm is caused by detector switching.

Native cable bacteria produced an absorption band at 410 nm, which is in the region where we expect Soret bands of various types of cytochromes (Figure 8). The broader and weaker Q band of cytochromes (~530 nm) was not detected (Azarkina et al., 1997; Oellerich et al., 2002; Mugnol et al., 2008; Diuba et al., 2023). Cytochrome signals were absent in fiber skeletons, as was the case for the Raman spectra, confirming that cytochromes are removed during the fiber skeleton extraction procedure (Meysman et al., 2019; Boschker et al., 2021). Both native cable bacteria and fiber skeletons yielded two absorption bands at 224 nm and 266 nm that likely originate from aromatic amino acids like tryptophan, phenyl alanine, and tyrosine, which have strong absorption bands in the UV region (Held, 2003). Fiber skeletons exhibited a weak and broad absorption band between 400 and 500 nm that could be caused by absorption from the Ni-cofactor. No absorption bands were observed in the NIR region, even though the Raman spectra suggest that the Ni-cofactor displays resonance at these wavelengths. This is most likely a problem of sensitivity. The Ni-cofactor is present at relatively low levels in fiber skeletons [Ni concentration < 1 p.p.t.; Boschker et al. (2021)], which is sufficient for detection by resonance Raman microscopy, but not by UV-Vis-NIR absorption spectroscopy.

## 4 Discussion

### 4.1 The Raman fingerprint of native cable bacterium filaments

Raman microscopy has a rich history in the field of microbiology due to its ability to provide a chemical “fingerprint” that can differentiate between different microbial taxa, enabling label-free detection and identification (Jarvis and Goodacre, 2004; Harz et al., 2009; de Siqueira e Oliveira et al., 2012; Witkowska et al., 2018). In addition, Raman spectra can provide biochemical information at the single-cell level, which enables insight into the metabolism and physiological state of a bacterial cell (Berg et al., 2014; Oren et al., 2015; Cui et al., 2018). The Raman fingerprint of bacteria is comprised of peaks originating from proteins, lipids, carbohydrates, and nucleic acids (Huang et al., 2010). In some cases, intense peaks are produced by chromophores in microbial pigments, like carotenoids and chlorophylls, which can exhibit greatly enhanced scattering (Jehlička et al., 2014). Here, we demonstrate that Raman microscopy with multiple wavelengths provides a non-destructive way to gain insight into the biochemical make-up of cable bacteria. By analyzing cable bacteria with different laser wavelengths, we selectively enhance Raman signals of cytochromes and the recently identified Ni-cofactor, but also detect various other cell constituents, like general proteins and polyphosphate granules.

The blue (405 nm) and green (532 nm) Raman spectra demonstrate that cytochromes are abundantly present in native cable bacterium filaments (Figure 3), as suggested by the presence of cytochrome genes in genomic data of cable bacteria (Kjeldsen et al., 2019). Cytochrome signals were also observed in earlier Raman microscopy analyses of native cable bacteria by Bjerg et al. (2018), Meysman et al. (2019), and Boschker et al. (2021). Cytochromes play a vital role in extracellular electron transport in metal-reducing bacteria like *Geobacter* and *Shewanella*. In *Shewanella*, multi-heme cytochromes are abundant in the membrane extensions that support extracellular electron transport (El-Naggar et al., 2010), while *Geobacter* produces conductive nanowires with tightly stacked, multi-heme cytochromes for transport to extracellular electron acceptors (Subramanian et al., 2018; Wang et al., 2019). However, the analysis here confirms that cytochromes are not central to long-distance electron transport in cable bacteria. Comparing the blue (405 nm) and green (532 nm) spectra in Figures 3, 4, our data reveal that cytochromes are removed upon the extraction of fiber skeletons from native cable bacteria since cytochrome-related peaks disappear. Fiber skeletons retain the periplasmic fibers that convey the electrical currents, and therefore, cytochromes cannot make up the conductive fibers (Meysman et al., 2019). The cytochrome signals observed in native cable bacteria (Figure 3) must thus link to cytochromes involved in physiological processes other than long-distance electron transport. We hypothesize that cytochromes could play an auxiliary role in long-distance electron transport. Raman microscopy has previously demonstrated that the oxidation state of cytochromes displays a gradient along active, conducting cable bacterium filaments (Bjerg et al., 2018). Moreover, cytochromes immediately change to a more reduced state when oxygen is removed or filaments are cut, indicating that there is a clear connection between the cytochrome oxidation state and conduction (Bjerg et al., 2018). We speculate that periplasmatic cytochromes could be involved in the up- and downloading of electrons to and from the conductive fibers. Alternatively, cytochromes could function as capacitors, providing a temporary storage for electrons when suitable electron acceptors (oxygen, nitrate) are temporarily unavailable (Esteve-Núñez et al., 2008), or perhaps, cytochromes could sense the potential of the conductive fibers to control chemotaxis (Bjerg et al., 2023).

Various protein-related Raman signals, like the Amide I mode (1,660 cm^−1^) and Phe ring breathing (1,004 cm^−1^), were detected in native cable bacteria, along with the CH_2_ scissoring (1,450 cm^−1^) and C-H stretching modes (2,940 cm^−1^), which are generally present in lipids, proteins, carbohydrates, and nucleic acids. Contrary, the signals of these general cellular compounds decreased strongly in the Raman spectra of fiber skeletons. After extraction, we only detected strong Raman signals from the Ni-cofactor and very weak protein signals (Amide I, 1,660 cm^−1^) in the red (785 nm) spectrum (Figure 4). This observation confirms that most of the cell content and membranes was removed during the SDS/EDTA extraction procedure, and that the remaining structure is enriched in the Ni-cofactor. Since fiber skeletons are made up of the conductive fibers superimposed upon a thin, carbohydrate-rich sheath (Boschker et al., 2021), it is likely the residual protein signal originates primarily from the fibers.

Phosphate-related signals (~980 cm^−1^; Frost et al., 2016; Nkebiwe et al., 2022) were detected in some Raman spectra of native cable bacteria (Figure 3) and fiber skeletons (Figure 4). This indicates that polyphosphate granules were occasionally present in cable bacterium cells. Polyphosphate granules are produced as a form of energy storage by active cable bacterium cells with access to sulfide, and are consumed to power the basal metabolism when cells reside in the oxygen-rich, sulfide-free top layer of the sediment (Sulu-Gambari et al., 2016b; Geerlings et al., 2022). Since cable bacterium filaments were mostly harvested from the oxic top layer of the sediment, polyphosphate granules were only sporadically detected. Large polyphosphate granules have also been observed in fiber skeletons (Cornelissen et al., 2018) and are likely obstructed from washing out during the extraction procedure by the carbohydrate sheath and conductive fibers.

A remarkable feature in the Raman spectra of native filaments are the two low-frequency peaks that form the core of the Ni-cofactor’s Raman fingerprint (Boschker et al., 2021). These two Raman active modes are prominently present in both native cable bacteria (372 and 492 cm^−1^) and fiber skeletons (367 and 496 cm^−1^) when probed with all laser sources (Figures 3, 4). This Raman signature is present in different morphotypes of cable bacteria retrieved from various freshwater and marine environments, implying that the Ni-cofactor is universally present in cable bacteria (Boschker et al., 2021). When screening the Raman signatures produced by other micro-organisms (de Siqueira e Oliveira et al., 2012; Oren et al., 2015; Serrano et al., 2015; Wolf et al., 2018; Bin et al., 2019; Ho et al., 2019), the two Ni-cofactor peaks are never present, which suggests that the Ni-cofactor is specific to cable bacteria. Additionally, the Ni-cofactor’s Raman signal is intense across a broad range of wavelengths (405–1,064 nm; Figures 3, 4). Consequently, it provides a clear and distinct signature that enables us to distinguish cable bacteria easily and reliably from other bacteria with Raman microscopy. Non-destructive, fast microbial phenotyping using Raman microscopy is an active field of research (Prucek et al., 2012; Li et al., 2013; Oren et al., 2015; Colniţă et al., 2017; Witkowska et al., 2018; Ho et al., 2019), so the existence of a unique and intense Raman fingerprint facilitates the detection of cable bacteria in complex environmental samples.

### 4.2 The orientation dependence of the vibrational modes in the Ni-cofactor

Our spectroscopic investigations provide a more detailed picture of the Ni-cofactor unique to cable bacteria. Raman spectra recorded at different wavelengths (Figure 4) and analysis with an ultra-low frequency filter set (Figure 7) enabled the identification of multiple characteristic Ni-cofactor modes, in addition to the two low-frequency modes (372 and 492 cm^−1^) that were previously known (Table 2). This way, we have identified a total of 13 Raman active modes that make up the Raman fingerprint of the Ni-cofactor. The commonality is that most of these modes display a strong orientation-dependent signal response, which enables to selectively distinguish the modes of the Ni-cofactor from those of other biomass compounds.

The orientation-dependent behavior of the Ni-cofactor modes is a form of anisotropy. Anisotropy in Raman spectroscopy manifests itself as the selective enhancement or suppression of Raman scattering from vibrational modes determined by the molecular and/or structural symmetry of a material. It is common in crystals and highly structured biological materials, which produce more intense Raman signals when light polarized along a crystal or orientation axis interacts with molecular vibrations in the same plane (Loudon, 1964). Prime examples are collagen bundles (Janko et al., 2010), protein sheets in feathers, pigments in the eyespot of *Chlamydomonas* (Tsuboi, 2002), and helical proteins in viruses (Tsuboi et al., 2000) because these systems comprise molecular structures with a high degree of directionality necessary for their biological function.

The observed Raman anisotropy in the conductive fibers of cable bacteria is substantial. The intensity of the five signature Ni-cofactor modes (367, 496, 1,146, 1,181, and 1,219 cm^−1^) is a factor 2 to 6 higher when fibers are oriented parallelly to the polarization of the incident light (Figure 6; Supplementary Figure 1). The (ultra)low-frequency modes (95, 121, 182, and 278 cm^−1^) show similar, but smaller anisotropic behavior (Figure 7). This signal increase is likely caused by enhanced polarizability along the orientation axis (i.e., long axis) of the Ni-cofactor as observed in planar molecules aligned on thin films (Kowalska et al., 2012). Therefore, the observed anisotropy indicates that the cofactor likely constitutes a planar molecule, oriented with its long axis parallel to the direction of the conductive fibers and thus the path of electron transport. This observation is consistent with the proposition that the Ni-cofactor is a nickel bis(1,2-dithiolene) complex, as further discussed below.

### 4.3 The Raman fingerprint of the Ni-cofactor

Curiously, the Raman fingerprint of the Ni-cofactor shows little to no resemblance to other known Ni-containing cofactors in biology. For instance, the Raman spectrum of Ni superoxide dismutase (NiSOD) has two Ni-S stretching modes (349 and 365 cm^−1^) involving two coordinating cysteine residues, but no feature around 490 cm^−1^ (Fiedler et al., 2005). On the other hand, the Raman spectrum of NiFe hydrogenase is far more complicated than that of the Ni-cofactor, containing peaks linked to the presence of 4Fe-4S clusters and Fe-CO/-CN moieties in the catalytic center (Horch et al., 2014). Previous studies have also shown that iron is not present in the conductive fibers (Boschker et al., 2021), so the cofactor in cable bacteria is clearly distinct from that in NiFe hydrogenase. Finally, the cofactors in urease, CO dehydrogenase, and methyl-coenzyme M reductase lack Ni-S bonds (Tang et al., 2002; Can et al., 2014; Chauhan et al., 2020), and therefore share no structural homology.

The Ni-cofactor’s Raman fingerprint also stands out because of the intensity of the two low-frequency modes (372 and 492 cm^−1^), which overshadow the signals of other general cellular compounds like proteins. This is remarkable since the cofactor only constitutes a miniscule fraction of the total cable bacterium biomass. Fiber skeletons are enriched in Ni-cofactors after most of the cell material is removed during SDS/EDTA extraction. However, the Ni content in fiber skeletons, which is directly representative of the Ni-cofactor, amounts to less than 0.05 atom%, while proteins and polysaccharides make up most of the fiber skeleton mass (Boschker et al., 2021). And yet, the cofactor’s Raman signals dominate the fiber skeleton spectra. As it happens, the cofactor’s Raman signals are of the same magnitude as those of cytochromes in native cable bacteria, which are likely more abundant (Bjerg et al., 2018; Kjeldsen et al., 2019) and exhibit strong Raman scattering at 405 and 532 nm. Consequently, the intense Raman signals of the Ni-cofactor across the entire range of tested wavelengths are likely the result of resonant Raman scattering. Yet, this hypothesis could not be substantiated by UV-Vis-NIR absorption measurements (Figure 8), likely because of the low concentration of the Ni-cofactor, and so the technique is not sensitive enough to detect any cofactor-related absorption in the examined samples. Accordingly, further UV-Vis-NIR investigations are needed to verify the resonant character.

Due to the absence of biological analogs, we expanded the search for potential structures to include abiotic sulfur-coordinated nickel complexes. Nickel bis(1,2-dithiolene) complexes have been extensively investigated in organic chemistry and have previously been proposed as potential analogs for the Ni-cofactor by Boschker et al. (2021). The two dithiolene ligands form a planar five-member ring with a nickel center (Schrauzer and Mayweg, 1962). These planar, transition metal complexes produce Raman spectra that indeed resemble the spectra of the Ni-cofactor in cable bacteria. Raman active modes in nickel bis(1,2-dithiolene) complexes include S-Ni-S deformation (~180 cm^−1^), asymmetric Ni-S stretching (~280 cm^−1^), symmetric Ni-S stretching (~340 cm^−1^), C-S stretching coupled to ring deformation (~500 cm^−1^), and stretching in sulfur-based radicals (1,100–1,200 cm^−1^; Johnson, 2004; Petrenko et al., 2006; Ray et al., 2007). The wavenumbers and relative intensity of these modes are nearly identical to those in the Ni-cofactor (Figure 4; Supplementary Table 3). However, there are small discrepancies in the exact peak positions and the number of peaks, which could imply slight structural differences (e.g., bond length and/or bond angle) or indicate that the exact molecular structure of the coordinating dithiolene ligands differ. Nevertheless, we propose an annotation of the Ni-cofactor’s Raman active vibrational modes based on the work on nickel bis(1,2-dithiolene) complexes by Johnson (2004) and Petrenko et al. (2006).

Assuming a nickel bis(1,2-dithiolene)-like structure, we were able to annotate most of the Ni-cofactor’s modes (Johnson, 2004; Petrenko et al., 2006). Two low-frequency modes (367 and 496 cm^−1^) form the core of the Ni-cofactor’s Raman fingerprint. We observed these two intense modes in all Raman spectra of both native cable bacteria and fiber skeletons (Figures 3, 4) and assigned them to symmetric Ni-S stretching (367 cm^−1^) and C-S stretching coupled to a ring deformation (496 cm^−1^; Johnson, 2004). In nickel bis(1,2-dithiolene) complexes, these modes also dominate the Raman spectrum because of resonance enhanced scattering (Ray et al., 2007). Isotopic labeling with either ^13^C (this work; Figure 5) or ^34^S (Boschker et al., 2021) caused a downward shift of the 496 cm^−1^ peak, confirming the C-S origin of this peak. Furthermore, the split from the single 496 cm^−1^ peak into two peaks (483 and 491 cm^−1^) upon ^13^C-labeling suggests this molecular vibration is not a pure C-S stretching mode, but rather C-S stretching coupled to the deformation of the dithiolene ring structure (Johnson, 2004). The two “core” modes also exhibited a peak shift due to the SDS/EDTA extraction procedure. The Ni-S stretching mode (367 cm^−1^) is shifted downwards (−5 cm^−1^), indicating a slight increase in bond length. The 496 cm^−1^ mode is shifted upwards (+4 cm^−1^), which suggests that the C-S and/or C=C bonds of the ligands slightly shorten. In nickel bis(1,2-dithiolene) complexes, the determination of oxidation state is not obvious due to the non-innocent character of the dithiolene ligands (Velho et al., 2021). Non-innocence implies that the electron density is not mainly localized on the central metal atom, but instead distributed over all the atoms of the molecule. A change in the electronic structure does therefore not change the planar geometry but can affect the bond lengths and angles within the complex (Velho et al., 2021), which is then reflected in the Raman spectrum. The SDS/EDTA treatment could affect the electronic structure of the Ni-cofactor through conformation changes of the surrounding proteins induced by SDS or through chelation of Ni atoms by EDTA. Currently however, we do not know how Ni-cofactors are embedded in the protein structure of the fibers, so the actual structural changes upon extraction remain uncertain.

We also detected a group of three prominent peaks in the mid-frequency region (1,146, 1,181, and 1,219 cm^−1^) upon irradiation with longer wavelengths (633, 785, and 1,064 nm). Previously, these “castle” peaks were assigned to stretching in C-C or C-N ligands of the Ni center (Boschker et al., 2021). However, we concur that this assignment was incorrect, and instead these peaks originate from C-S stretching in sulfur-based radicals (C=S·) in the dithiolene ligands (Petrenko et al., 2006). These bonds are selectively enhanced due to an intervalence charge transfer (IVCT) band in the near-infrared region, explaining why the castle is only detected in the orange, red, and infrared spectrum (Ray et al., 2007).

In addition to the five modes already discussed, we observed eight (ultra)low-frequency modes that produced weak Raman signals. These modes are most clearly seen in the ULF Raman spectrum of fiber skeletons (Figure 7) and include a S-Ni-S deformation mode (182 cm^−1^), an asymmetric Ni-S stretching mode (278 cm^−1^), and six modes (53, 95, 121, 308, 448_,_ and 622 cm^−1^) that could not be confidently assigned (Table 2). Like their more intense counterparts, the 95, 121, 182, and 278 cm^−1^ mode also display an orientation-dependent signal response. This allows us to unambiguously assign these unannotated, low-frequency modes to the Ni-cofactor. Since the frequency of molecular vibrations is determined by the atomic mass and bond strength (Nakamoto, 2008), the ultralow-frequency modes (50–200 cm^−1^) likely constitute vibrations of the entire coordinating ligands in the cofactor. Together, these 13 Raman active modes make up the Raman fingerprint of the Ni-cofactor (Table 2).

The strong response of the two low-frequency modes (367 cm^−1^ and 496 cm^−1^) across the range of tested wavelengths (Figure 4) is highly unusual for a biological material. Other Ni metalloproteins, like superoxide dismutase, [NiFe]-hydrogenase, CO dehydrogenase, and lactate racemase exclusively exhibit absorption related to their Ni-containing cofactor in the region between 325 and 450 nm (Bonams and Luddent, 1987; Ryan and Maroney, 2013; Xu et al., 2017; Caserta et al., 2020). A nickel bis(1,2-dithiolene)-like structure could explain this behavior, as these complexes are strong chromophores that exhibit broad and intense absorption ranging from the UV to the NIR region. Absorption across this broad range stems from a variety of possible electronic transitions and charge transfers. The 1,2-dithiolene ligands enable intense ligand-to-metal charge transfer bands, intra-ligand, and π – π* transitions below 400 nm (Matsubayashi et al., 1992; Ray et al., 2005). Intense absorption in the NIR region results from π – π* transitions in ligand-centered orbitals (Mueller-Westerhoff et al., 1991). In some complexes, ligand-to-ligand (LLCT) and intervalence charge transfer bands (IVCT) yield additional absorption in the NIR region (Ray et al., 2005).

Even though the observed Raman scattering of the Ni-cofactor indicates it should possess distinct electronic transitions and charge-transfer bands, we were unable to detect the corresponding absorption peaks. This is likely the result of the low concentration of the Ni-cofactor in native cable bacterium filaments (Boschker et al., 2021), and the lack of sensitivity of UV-Vis-NIR spectroscopy. More concentrated samples are challenging to obtain as we manually harvest and process individual filaments to produce cable bacterium biomass. Therefore, for the current samples, absorption spectroscopy probably lacks the sensitivity to detect absorbance by the cofactor. For reference, cytochromes, which are far more abundant in cable bacteria (Bjerg et al., 2018; Kjeldsen et al., 2019), only produce a small peak at their absorption maximum (Soret band, 410 nm; Figure 8). The detection of absorption in the NIR region is further hampered by noise caused by detector switching in a crucial range (800–900 nm), the strong absorption of water, and potentially an increase in Rayleigh and Mie scattering, resulting in a decreasing baseline signal (Young, 1981; Bassan et al., 2009). The biomass limitation could be overcome in future experiments by identifying the biosynthesis pathway of the Ni-cofactor and overexpression in a model organism. This way, one could produce and purify large quantities of Ni-cofactor and use concentrated solutions for UV-Vis-NIR absorption and other spectroscopy techniques to further unravel its molecular structure.

Multi-wavelength Raman microscopy has improved our understanding of the biochemical composition of cable bacterium filaments, while a combination of stable isotope labeling, orientation-dependent and ULF Raman microscopy have shed light on the structure and organization of a novel Ni-cofactor. Combining these techniques, we identified a unique spectroscopic fingerprint with which cable bacteria can easily be distinguished from other bacteria. This fingerprint is attributed to the strong Raman signals produced by the novel Ni-cofactor and features two vibrational modes (Ni-S stretching—367 cm^−1^, C-S stretching + ring deformation—496 cm^−1^) that stand out due to their remarkable intense Raman signal across a broad spectral range (405–1,064 nm). Next to these, we observed 11 other vibrational modes that can be unambiguously linked to the Ni-cofactor. None of the other Ni-containing cofactors currently known to biology produce similar Raman signatures to that in the conductive fibers of cable bacteria (Shiemke et al., 1983; Qiu et al., 1996; Fiedler et al., 2005; Horch et al., 2014; Chauhan et al., 2020). Therefore, it seems cable bacteria produce a Ni-cofactor with a unique molecular structure that is new in the field of biology.

The vibrational modes that make up the Raman fingerprint of the Ni-cofactor are strikingly similar to those observed in nickel bis(1,2-dithiolene) complexes, suggesting that the cofactor must share a high degree of structural analogy with these complexes. Still, several aspects require further study, as its exact molecular structure remains unclear. Furthermore, little is known about the genes and proteins involved in the synthesis of the Ni-cofactor, and its subsequent assembly into the protein matrix of the conductive fibers. All these factors are crucial to assemble the Ni-based, long-range electron transport pathway that we characterize in this work.

## Data availability statement

The datasets presented in this study can be found in online repositories. The names of the repository/repositories and accession number(s) can be found at: https://doi.org/10.5281/zenodo.10285831.

## Author contributions

FM and HB designed the study. SH-M performed filament cultivation. SH-M and BS performed the filament isolation and fiber skeleton extraction. SH-M performed SEM image collection. BS, HB, GN, and MW performed Raman microscopy data collection. BS performed Raman data processing and analysis. HB first noted the dependence of Raman signal intensity on the orientation of the laser light polarization. KW and GN, respectively, coordinate and operate the Renishaw Raman infrastructure utilized at UAntwerpen, while MW operates the Horiba Raman infrastructure utilized at the Materials Characterization Lab of the Materials Research Institute at Pennsylvania State University. BS, HB, and GL performed UV-Vis-NIR absorption spectroscopy experiments. LP performed NanoSIMS scans and assisted BS with NanoSIMS data processing. All authors contributed to the article and approved the submitted version.

## References

[ref2] Alfano M. Cavazza C. (2020). Structure, function, and biosynthesis of nickel-dependent enzymes. Protein Sci. 29, 1071–1089. doi: 10.1002/pro.3836, PMID: 32022353 PMC7184782

[ref3] Azarkina N. Borisov V. Konstantinov A. A. (1997). Spontaneous spectral changes of the reduced cytochrome bd. FEBS Lett. 416, 171–174. doi: 10.1016/S0014-5793(97)01196-4, PMID: 9369207

[ref4] Bassan P. Byrne H. J. Bonnier F. Lee J. Dumas P. Gardner P. (2009). Resonant Mie scattering in infrared spectroscopy of biological materials – understanding the ‘dispersion artefact’. Analyst 134, 1586–1593. doi: 10.1039/b904808a, PMID: 20448924

[ref5] Berg J. S. Schwedt A. Kreutzmann A. C. Kuypers M. M. M. Milucka J. (2014). Polysulfides as intermediates in the oxidation of sulfide to sulfate by Beggiatoa spp. Appl. Environ. Microbiol. 80, 629–636. doi: 10.1128/AEM.02852-13, PMID: 24212585 PMC3911116

[ref6] Bin T. Shaofeng Z. Yi W. Beiping Z. Lihua Z. Yong Y. (2019). Molecular insight into electron transfer properties of extracellular polymeric substances of electroactive bacteria by surface-enhanced Raman spectroscopy. Sci. China Technol. Sci. 62, 1679–1687. doi: 10.1007/s11431-018-9437-0

[ref7] Bjerg J. Boschker H. T. S. Larsen S. Berry D. Schmid M. Millo D.. (2018). Long-distance electron transport in individual, living cable bacteria. Proc. Natl. Acad. Sci. U. S. A. 115, 5786–5791. doi: 10.1073/pnas.1800367115, PMID: 29735671 PMC5984516

[ref8] Bjerg J. Lustermans J. Marshall I. Mueller A. Brokjær S. Thorup C.. (2023). Cable bacteria with electric connection to oxygen attract flocks of diverse bacteria. Nat. Commun. 14, 1–8. doi: 10.1038/s41467-023-37272-8 36959175 PMC10036481

[ref9] Bonams D. Luddent P. W. (1987). Purification and characterization of carbon monoxide dehydrogenase, a nickel, zinc, iron-Sulfur protein, from *Rhodospirillum rubrum*. J. Biol. Chem. 262, 2980–2987. doi: 10.1016/S0021-9258(18)61456-5, PMID: 3029096

[ref10] Boschker H. T. S. Cook P. L. M. Polerecky L. Eachambadi R. T. Lozano H. Hidalgo-Martinez S.. (2021). Efficient long-range conduction in cable bacteria through nickel protein wires. Nat. Commun. 12, 1–30. doi: 10.1038/s41467-021-24312-4 34183682 PMC8238962

[ref12] Can M. Armstrong F. A. Ragsdale S. W. (2014). Structure, function, and mechanism of the nickel metalloenzymes, CO dehydrogenase, and acetyl-CoA synthase. Chem. Rev. 114, 4149–4174. doi: 10.1021/cr400461p, PMID: 24521136 PMC4002135

[ref13] Caserta G. Lorent C. Ciaccafava A. Keck M. Breglia R. Greco C.. (2020). The large subunit of the regulatory [NiFe]-hydrogenase from *Ralstonia eutropha* – a minimal hydrogenase? Chem. Sci. 11:5453. doi: 10.1039/D0SC01369B, PMID: 34094072 PMC8159394

[ref14] Chassé M. Lelong G. Nijnatten P. Schoofs I. Wolf J. Galoisy L.. (2015). Optical absorption microspectroscopy (μ-OAS) based on Schwarzschild-type Cassegrain optics. Appl. Spectrosc. 69, 457–463. doi: 10.1366/14-07628, PMID: 25741926

[ref15] Chauhan M. Jha A. Subramanian V. Valdastri P. (2020). pH characterization of urease using Raman Spectroscopy. in *Optics InfoBase Conference Papers* (Optica Publishing Group).

[ref16] Colniţă A. Dina N. E. Leopold N. Vodnar D. C. Bogdan D. Porav S. A.. (2017). Characterization and discrimination of gram-positive bacteria using raman spectroscopy with the aid of principal component analysis. Nanomaterials 7, 28–31. doi: 10.3390/nano7090248 28862655 PMC5618359

[ref17] Cornelissen R. Bøggild A. Thiruvallur Eachambadi R. Koning R. I. Kremer A. Hidalgo-Martinez S.. (2018). The cell envelope structure of cable bacteria. Front. Microbiol. 9:3044. doi: 10.3389/fmicb.2018.03044, PMID: 30619135 PMC6307468

[ref18] Cui L. Yang K. Li H. Z. Zhang H. Su J. Q. Paraskevaidi M.. (2018). Functional single-cell approach to probing nitrogen-fixing bacteria in soil communities by resonance Raman spectroscopy with 15N2 Labeling. Anal. Chem. 90, 5082–5089. doi: 10.1021/acs.analchem.7b05080, PMID: 29557648

[ref19] Czamara K. Majzner K. Pacia M. Z. Kochan K. Kaczor A. Baranska M. (2015). Raman spectroscopy of lipids: a review. J. Raman Spectrosc. 46, 4–20. doi: 10.1002/jrs.4607, PMID: 37769851

[ref20] Czernuszewicz R. S. (1993). “Resonance Raman spectroscopy of metalloproteins using CW laser excitation” in Methods of molecular biology (United States of America: Humana Press), 345–374. 10.1385/0-89603-215-9:345 21400146

[ref21] De Siqueira e Oliveira F. S. Giana H. E. Silveira L. (2012). Discrimination of selected species of pathogenic bacteria using near-infrared Raman spectroscopy and principal components analysis. J. Biomed. Opt. 17:107004. doi: 10.1117/1.JBO.17.10.107004, PMID: 23052563

[ref22] Diuba A. V. Vygodina T. V. Azarkina N. V. Arutyunyan A. M. Soulimane T. Vos M. H.. (2023). Individual heme a and heme a3 contributions to the Soret absorption spectrum of the reduced bovine cytochrome c oxidase. Biochim. Biophys. Acta 1864:148937. doi: 10.1016/j.bbabio.2022.148937, PMID: 36403793

[ref23] El-Naggar M. Y. Wanger G. Leung K. M. Yuzvinsky T. D. Southam G. Yang J.. (2010). Electrical transport along bacterial nanowires from *Shewanella oneidensis* MR-1. Proc. Natl. Acad. Sci. U. S. A. 107, 18127–18131. doi: 10.1073/pnas.1004880107, PMID: 20937892 PMC2964190

[ref24] Esteve-Núñez A. Sosnik J. Visconti P. Lovley D. R. (2008). Fluorescent properties of c-type cytochromes reveal their potential role as an extracytoplasmic electron sink in *Geobacter sulfurreducens*. Environ. Microbiol. 10, 497–505. doi: 10.1111/j.1462-2920.2007.01470.x, PMID: 18093163

[ref25] Ferraro J. R. Nakamoto K. Brown C. W. (2003). Introductory Raman spectroscopy. 2nd Edn Elsevier Inc. Amsterdam, The Netherlands.

[ref26] Fiedler A. T. Bryngelson P. A. Maroney M. J. Brunold T. C. (2005). Spectroscopic and computational studies of Ni superoxide dismutase: electronic structure contributions to enzymatic function. J. Am. Chem. Soc. 127, 5449–5462. doi: 10.1021/ja042521i, PMID: 15826182

[ref27] Fontecilla-Camps J. C. (2022). Nickel and the origin and early evolution of life. Metallomics 14:mfac016. doi: 10.1093/mtomcs/mfac016, PMID: 35294026

[ref28] Freire P. T. C. Barboza F. M. Lima J. A. Melo F. E. A. Filho J. M. (2017). Raman spectroscopy of amino acid crystals London, United Kingdom: IntechOpen.

[ref29] Frost R. L. Scholz R. López A. (2016). A Raman and infrared spectroscopic study of the phosphate mineral laueite. Vib. Spectrosc. 82, 31–36. doi: 10.1016/j.vibspec.2015.12.001, PMID: 37394288

[ref30] Garton S. D. Hilton J. Oku H. Crouse B. R. Rajagopalan K. V. Johnson M. K. (1997). Active site structures and catalytic mechanism of *Rhodobacter sphaeroides* dimethyl sulfoxide reductase as revealed by resonance Raman spectroscopy. J. Am. Chem. Soc. 119, 12906–12916. doi: 10.1021/ja972109l

[ref31] Geelhoed J. Thorup C. Bjerg J. Schreiber L. Nielsen L. P. Schramm A.. (2023). Indications for a genetic basis for big bacteria and description of the giant cable bacterium Candidatus Electrothrix gigas sp. nov. Microbiol Spectr 11:e0053823. doi: 10.1128/spectrum.00538-23, PMID: 37732806 PMC10580974

[ref32] Geerlings N. M. J. Karman C. Trashin S. As K. S. Kienhuis M. V. M. Hidalgo-Martinez S.. (2020). Division of labor and growth during electrical cooperation in multicellular cable bacteria. Proc. Natl. Acad. Sci. U. S. A. 117, 5478–5485. doi: 10.1073/pnas.1916244117, PMID: 32094191 PMC7071850

[ref33] Geerlings N. M. J. Kienhuis M. V. M. Hidalgo-Martinez S. Hageman R. Vasquez-Cardenas D. Middelburg J. J.. (2022). Polyphosphate dynamics in cable bacteria. Front. Microbiol. 13:1641. doi: 10.3389/fmicb.2022.883807 PMC9159916 35663875

[ref34] Han S. Czernuszewicz R. S. Kimura T. Adams M. W. W. Spiro T. G. (1989). Fe2S2 protein resonance Raman spectra revisited: structural variations among Adrenodoxin, ferredoxin, and red paramagnetic protein. J. Am. Chem. Soc. 111, 3505–3511. doi: 10.1021/ja00192a003

[ref35] Harz M. Rösch P. Popp J. (2009). Vibrational spectroscopy-A powerful tool for the rapid identification of microbial cells at the single-cell level. Cytometry Part A 75, 104–113. doi: 10.1002/cyto.a.20682 19156822

[ref37] Hassing S. Sonnich Mortensen O. (1980). Kramers–Kronig relations and resonance Raman scattering. J. Chem. Phys. 73, 1078–1083. doi: 10.1063/1.440280

[ref38] Held P. (2003). Peptide and amino acid quantification using UV fluorescence in synergy HT multi-mode microplate reader. BioTek, 1–8. Available at: www.biotek.com (Accessed 11 January 2023).

[ref39] Ho C. S. Jean N. Hogan C. A. Blackmon L. Jeffrey S. S. Holodniy M.. (2019). Rapid identification of pathogenic bacteria using Raman spectroscopy and deep learning. Nat. Commun. 10:9. doi: 10.1038/s41467-019-12898-9, PMID: 31666527 PMC6960993

[ref40] Horch M. Schoknecht J. Mroginski M. A. Lenz O. Hildebrandt P. Zebger I. (2014). Resonance Raman spectroscopy on [NiFe] hydrogenase provides structural insights into catalytic intermediates and reactions. J. Am. Chem. Soc. 136, 9870–9873. doi: 10.1021/ja505119q, PMID: 24956459

[ref42] Hu S. Spiro T. G. Morris I. K. Singh J. P. Smith K. M. (1993). Complete assignment of cytochrome c resonance Raman spectra via enzymatic reconstitution with isotopically Labeled Hemes. J. Am. Chem. Soc. 115, 12446–12458. doi: 10.1021/ja00079a028

[ref43] Huang W. E. Li M. Jarvis R. M. Goodacre R. Banwart S. A. (2010). Shining light on the microbial world the application of Raman microspectroscopy. 1st Edn. Amsterdam, The Netherlands: Elsevier Inc. 10.1016/S0065-2164(10)70005-8 20359457

[ref44] Janko M. Davydovskaya P. Bauer M. Zink A. Stark R. W. (2010). Anisotropic Raman scattering in collagen bundles. Opt. Lett. 35:2765. doi: 10.1364/OL.35.002765, PMID: 20717450

[ref45] Jarvis R. M. Goodacre R. (2004). Ultra-violet resonance Raman spectroscopy for the rapid discrimination of urinary tract infection bacteria. FEMS Microbiol. Lett. 232, 127–132. doi: 10.1016/S0378-1097(04)00040-0, PMID: 15033230

[ref46] Jehlička J. Edwards H. G. M. Oren A. (2014). Raman spectroscopy of microbial pigments. Appl. Environ. Microbiol. 80, 3286–3295. doi: 10.1128/AEM.00699-14, PMID: 24682303 PMC4018853

[ref47] Johnson M. K. (2004). “Vibrational spectra of dithiolene complexes,” in Dithiolene Chemistry: Synthesis, Properties, and Applications, ed. Stiefel E. (Hoboken, NJ, United States of America: John Wiley & Sons, Inc), 213–266. doi: 10.1002/0471471933.ch4

[ref48] Kengne-Momo R. P. Daniel P. Lagarde F. Jeyachandran Y. L. Pilard J. F. Durand-Thouand M. J.. (2012). Protein interactions investigated by the Raman spectroscopy for biosensor applications. Int J Spectrosc 2012, 1–7. doi: 10.1155/2012/462901, PMID: 36140152

[ref49] Kjeldsen K. U. Schreiber L. Thorup C. A. Boesen T. Bjerg J. T. Yang T.. (2019). On the evolution and physiology of cable bacteria. Proc. Natl. Acad. Sci. 116, 19116–19125. doi: 10.1073/pnas.1903514116, PMID: 31427514 PMC6754541

[ref50] Kowalska P. Cheeseman J. R. Razmkhah K. Green B. Nafie L. A. Rodger A. (2012). Experimental and theoretical polarized raman linear difference spectroscopy of small molecules with a new alignment method using stretched polyethylene film. Anal. Chem. 84, 1394–1401. doi: 10.1021/ac202432e, PMID: 22122486

[ref51] Li M. Huang W. E. Gibson C. M. Fowler P. W. Jousset A. (2013). Stable isotope probing and raman spectroscopy for monitoring carbon flow in a food chain and revealing metabolic pathway. Anal. Chem. 85, 1642–1649. doi: 10.1021/ac302910x, PMID: 23259452

[ref52] Liland K. H. Almøy T. Mevik B. H. (2010). Optimal choice of baseline correction for multivariate calibration of spectra. Appl. Spectrosc. 64, 1007–1016. doi: 10.1366/000370210792434350, PMID: 20828437

[ref53] Liu J. Chakraborty S. Hosseinzadeh P. Yu Y. Tian S. Petrik I.. (2014). Metalloproteins containing cytochrome, iron-sulfur, or copper redox centers. Chem. Rev. 114, 4366–4369. doi: 10.1021/cr400479b, PMID: 24758379 PMC4002152

[ref54] Liu C. Y. Webster D. A. (1974). Spectral characteristics and interconversions of the reduced, oxidized, and oxygenated forms of purified cytochrome o. J. Biol. Chem. 249, 4261–4266. doi: 10.1016/S0021-9258(19)42511-8, PMID: 4369203

[ref55] Loudon R. (1964). The Raman effect in crystals. Adv. Phys. 13, 423–482. doi: 10.1080/00018736400101051, PMID: 38407354

[ref56] Malkin S. Y. Rao A. M. F. Seitaj D. Vasquez-Cardenas D. Zetsche E. M. Hidalgo-Martinez S.. (2014). Natural occurrence of microbial Sulphur oxidation by long-range electron transport in the seafloor. ISME J. 8, 1843–1854. doi: 10.1038/ismej.2014.41, PMID: 24671086 PMC4139731

[ref57] Matsubayashi G. E. Tanaka S. Yokozawa A. (1992). Crystal structures of [nBun4]2[M(C3S5)(C 3Se5)] (M = Ni or pd) and properties of the nickel(II) complex. J. Chem. Soc. Dalton Trans. 20, 1827–1830. doi: 10.1039/DT9920001827

[ref58] Meysman F. J. R. Cornelissen R. Trashin S. Bonné R. Martinez S. H. van der Veen J.. (2019). A highly conductive fibre network enables centimetre-scale electron transport in multicellular cable bacteria. Nat. Commun. 10, 1–8. doi: 10.1038/s41467-019-12115-7 31511526 PMC6739318

[ref59] Milazzo L. Tognaccini L. Howes B. D. Smulevich G. (2018). Probing the non-native states of cytochrome c with resonance Raman spectroscopy: a tool for investigating the structure–function relationship. J. Raman Spectrosc. 49, 1041–1055. doi: 10.1002/jrs.5315

[ref60] Miura T. Thomas G. J. (1995). “Raman spectroscopy of proteins and their assemblies” in Proteins: Structure, function, and engineering. eds. Biswas B. B. Roy S. (Boston, MA: Springer US), 55. 10.1007/978-1-4899-1727-0_3 7900183

[ref61] Mueller-Westerhoff U. T. Vance B. Ihl Yoon D. (1991). The synthesis of dithiolene dyes with strong near-IR absorption. Tetrahedron 47, 909–932. doi: 10.1016/S0040-4020(01)80932-7

[ref62] Mugnol K. C. U. Ando R. A. Nagayasu R. Y. Faljoni-Alario A. Brochsztain S. Santos P. S.. (2008). Spectroscopic, structural, and functional characterization of the alternative low-spin state of horse heart cytochrome c. Biophys. J. 94:4066. doi: 10.1529/biophysj.107.116483, PMID: 18227133 PMC2367172

[ref63] Nakamoto K. (2008). “Theory of Normal Vibrations” in Infrared and Raman Spectra of Inorganic and Coordination Compounds, (Hoboken, NJ, United States of America: John Wiley & Sons, Inc.), 1–147. doi: 10.1002/9780470405840.ch1

[ref64] Nielsen L. P. Risgaard-Petersen N. Fossing H. Christensen P. B. Sayama M. (2010). Electric currents couple spatially separated biogeochemical processes in marine sediment. Nature 463, 1071–1074. doi: 10.1038/nature08790, PMID: 20182510

[ref65] Nkebiwe P. M. Sowoidnich K. Maiwald M. Sumpf B. Hartmann T. E. Wanke D.. (2022). Detection of calcium phosphate species in soil by confocal μ-Raman spectroscopy. J. Plant Nutr. Soil Sci. 185, 221–231. doi: 10.1002/jpln.202100233

[ref66] Oellerich S. Wackerbarth H. Hildebrandt P. (2002). Spectroscopic characterization of nonnative conformational states of cytochrome c. J. Phys. Chem. B 106, 6566–6580. doi: 10.1021/jp013841g, PMID: 29679799

[ref67] Oren A. Mana L. Jehlička J. (2015). Probing single cells of purple sulfur bacteria with Raman spectroscopy: carotenoids and elemental sulfur. FEMS Microbiol. Lett. 362:21. doi: 10.1093/femsle/fnv021 25682325

[ref68] Petrenko T. Ray K. Wieghardt K. E. Neese F. (2006). Vibrational markers for the open-shell character of transition metal bis-dithiolenes: an infrared, resonance Raman, and quantum chemical study. J. Am. Chem. Soc. 128, 4422–4436. doi: 10.1021/ja0578451, PMID: 16569020

[ref69] Pfeffer C. Larsen S. Song J. Dong M. Besenbacher F. Meyer R. L.. (2012). Filamentous bacteria transport electrons over centimetre distances. Nature 491, 218–221. doi: 10.1038/nature11586, PMID: 23103872

[ref70] Polerecky L. Adam B. Milucka J. Musat N. Vagner T. Kuypers M. M. M. (2012). Look@NanoSIMS—a tool for the analysis of nanoSIMS data in environmental microbiology. Environ. Microbiol. 14, 1009–1023. doi: 10.1111/j.1462-2920.2011.02681.x, PMID: 22221878

[ref71] Prescott B. Steinmetz W. Thomas G. J. (1984). Characterization of DNA structures by laser Raman spectroscopy. Biopolymers 23, 235–256. doi: 10.1002/bip.360230206, PMID: 6704487

[ref72] Prucek R. Ranc V. Kvítek L. Panáček A. Zbořil R. Kolář M. (2012). Reproducible discrimination between gram-positive and gram-negative bacteria using surface enhanced Raman spectroscopy with infrared excitation. Analyst 137, 2866–2870. doi: 10.1039/c2an16310a, PMID: 22577658

[ref73] Qiu D. Kumar M. Ragsdale S. W. Spiro T. G. (1996). Raman and infrared spectroscopy of cyanide-inhibited CO dehydrogenase/acetyl-CoA synthase from clostridium thermoaceticum: evidence for bimetallic enzymatic CO oxidation. J. Am. Chem. Soc. 118, 10429–10435. doi: 10.1021/ja960435f

[ref74] R Core Team (2018). R: A language and environment for statistical computing. R Foundation for Statistical Computing, Vienna, Austria. Available online at https://www.R-project.org/.

[ref75] Ray K. Petrenko T. Wieghardt K. Neese F. (2007). Joint spectroscopic and theoretical investigations of transition metal complexes involving non-innocent ligands. Dalton Trans., 1552–1566. doi: 10.1039/B700096K 17426855

[ref76] Ray K. Weyhermüller T. Neese F. Wieghardt K. (2005). Electronic structure of square planar bis(benzene-1,2-dithiolato)metal complexes [M(L)2]z (z = 2-, 1-0; M = Ni, pd, Pt, cu, au): an experimental, density functional, and correlated ab initio study. Inorg. Chem. 44, 5345–5360. doi: 10.1021/ic0507565, PMID: 16022533

[ref77] Risgaard-Petersen N. Kristiansen M. Frederiksen R. B. Dittmer A. L. Bjerg J. T. Trojan D.. (2015). Cable bacteria in freshwater sediments. Appl. Environ. Microbiol. 81, 6003–6011. doi: 10.1128/AEM.01064-15, PMID: 26116678 PMC4551263

[ref79] Ryan K. C. Maroney M. J. (2013). Nickel Superoxide Dismutase., 1st Edn, eds. Kretsinger R. Uversky V. Permyakov E.. New York, NY, United States of America: Springer. doi: 10.1007/978-1-4614-1533-6_84

[ref80] Salmaso B. L. Puppels G. J. Caspers P. J. Floris R. Wever R. Greve J. (1994). Resonance Raman microspectroscopic characterization of eosinophil peroxidase in human eosinophilic granulocytes. Biophys. J. 67, 436–446. doi: 10.1016/S0006-3495(94)80499-0, PMID: 7919017 PMC1225376

[ref81] Schrauzer G. N. Mayweg V. (1962). Reaction of diphenylacetylene with nickel Sulfides. J. Am. Chem. Soc. 5833:3221. doi: 10.1021/ja00875a061

[ref82] Serrano P. Hermelink A. Lasch P. de Vera J. P. König N. Burckhardt O.. (2015). Confocal Raman microspectroscopy reveals a convergence of the chemical composition in methanogenic archaea from a Siberian permafrost-affected soil. FEMS Microbiol. Ecol. 91:fiv126. doi: 10.1093/femsec/fiv126, PMID: 26499486

[ref83] Shafaat H. S. Weber K. Petrenko T. Neese F. Lubitz W. (2012). Key hydride vibrational modes in [NiFe] hydrogenase model compounds studied by resonance Raman spectroscopy and density functional calculations. Inorg. Chem. 51, 11787–11797. doi: 10.1021/ic3017276, PMID: 23039071

[ref84] Shiemke A. K. Dudley Eirich L. Loehr T. M. (1983). Resonance Raman spectroscopic characterization of the nickel cofactor, F430, form methanogenic bacteria. Biochim. Biophys. Acta 748, 143–147. doi: 10.1016/0167-4838(83)90037-7, PMID: 2738065

[ref85] Siamwiza M. N. Lord R. C. Chen M. C. Takamatsu T. Harada I. Matsuura H.. (1975). Interpretation of the doublet at 850 and 830 cm−1 in the Raman spectra of tyrosyl residues in proteins and certain model compounds. Biochemistry 14, 4870–4876. doi: 10.1021/bi00693a014, PMID: 241390

[ref86] Subramanian P. Pirbadian S. El-Naggar M. Y. Jensen G. J. (2018). Ultrastructure of *Shewanella oneidensis* MR-1 nanowires revealed by electron cryotomography. Proc. Natl. Acad. Sci. U. S. A. 115, E3246–E3255. doi: 10.1073/pnas.1718810115, PMID: 29555764 PMC5889646

[ref87] Sulu-Gambari F. Seitaj D. Behrends T. Banerjee D. Meysman F. J. R. Slomp C. P. (2016a). Impact of cable bacteria on sedimentary iron and manganese dynamics in a seasonally-hypoxic marine basin. Geochim. Cosmochim. Acta 192, 49–69. doi: 10.1016/j.gca.2016.07.028

[ref88] Sulu-Gambari F. Seitaj D. Meysman F. J. R. Schauer R. Polerecky L. Slomp C. P. (2016b). Cable bacteria control iron-phosphorus dynamics in sediments of a coastal Hypoxic Basin. Environ. Sci. Technol. 50, 1227–1233. doi: 10.1021/acs.est.5b04369 26720721

[ref89] Tang Q. Carrington P. E. Horng Y. C. Maroney M. J. Ragsdale S. W. Bocian D. F. (2002). X-ray absorption and resonance Raman studies of methyl-coenzyme M reductase indicating that ligand exchange and macrocycle reduction accompany reductive activation. J. Am. Chem. Soc. 124, 13242–13256. doi: 10.1021/ja020314h, PMID: 12405853

[ref90] Tsuboi M. (2002). Raman scattering anisotropy of biological systems. J. Biomed. Opt. 7:435. doi: 10.1117/1.1482720, PMID: 12175294

[ref91] Tsuboi M. Suzuki M. Overman S. A. Thomas G. J. (2000). Intensity of the polarized Raman band at 1340-1345 cm-1 as an indicator of protein α-helix orientation: application to Pf1 filamentous virus. Biochemistry 39, 2677–2684. doi: 10.1021/bi9918846, PMID: 10704218

[ref92] Velho M. F. G. Silva R. A. L. Belo D. (2021). The quest for single component molecular metals within neutral transition metal complexes. J Mater Chem C Mater 9, 10591–10609. doi: 10.1039/D1TC01407B

[ref93] Virdis B. Millo D. Donose B. C. Batstone D. J. (2014). Real-time measurements of the redox states of c-type cytochromes in electroactive biofilms: a confocal resonance Raman microscopy study. PLoS One 9:89918. doi: 10.1371/journal.pone.0089918, PMID: 24587123 PMC3934938

[ref94] Wang F. Gu Y. O’Brien J. P. Yi S. M. Yalcin S. E. Srikanth V.. (2019). Structure of microbial nanowires reveals stacked Hemes that transport electrons over micrometers. Cell 177, 361–369.e10. doi: 10.1016/j.cell.2019.03.029, PMID: 30951668 PMC6720112

[ref95] Wiercigroch E. Szafraniec E. Czamara K. Pacia M. Z. Majzner K. Kochan K.. (2017). Raman and infrared spectroscopy of carbohydrates: a review. Spectrochim. Acta A Mol. Biomol. Spectrosc. 185, 317–335. doi: 10.1016/j.saa.2017.05.045, PMID: 28599236

[ref96] Witkowska E. Korsak D. Kowalska A. Janeczek A. Kamińska A. (2018). Strain-level typing and identification of bacteria—a novel approach for SERS active plasmonic nanostructures. Anal. Bioanal. Chem. 410, 5019–5031. doi: 10.1007/s00216-018-1153-0, PMID: 29907950 PMC6061775

[ref97] Wolf M. W. Rizzolo K. Elliott S. J. Lehnert N. (2018). Resonance Raman, EPR and MCD spectroscopic investigation of Diheme cytochrome c peroxidases from Nitrosomonas europaea and *Shewanella oneidensis*. Biochemistry 57, 6416–6433. doi: 10.1021/acs.biochem.8b00732, PMID: 30335984 PMC6903397

[ref98] Xu T. Wodrich M. D. Scopelliti R. Corminboeuf C. Hu X. (2017). Nickel pincer model of the active site of lactate racemase involves ligand participation in hydride transfer. Proc. Natl. Acad. Sci. U. S. A. 114, 1242–1245. doi: 10.1073/pnas.1616038114, PMID: 28115700 PMC5307482

[ref99] Young A. T. (1981). Rayleigh scattering. Appl. Optics 20, 533–535. doi: 10.1364/AO.20.000533, PMID: 20309152

