# Supplementary material for: Multi-wavelength Raman microscopy of nickel-based electron transport in cable bacteria
Source: Front Microbiol. 2024 Mar 8;15:1208033. doi: 10.3389/fmicb.2024.1208033 (PMC10959288; doi:10.3389/fmicb.2024.1208033)

Supplementary Material

Multi-wavelength Raman microscopy of nickel-based electron transport in cable bacteria

Bent Smets^1*^, Henricus T. S. Boschker^1,2^, Maxwell T. Wetherington^3,^ Gérald Lelong^4^, Silvia Hidalgo-Martinez^1^, Lubos Polerecky^5^, Gert Nuyts^6,7^, Karolien De Wael^6^, Filip J. R. Meysman^1,2*^

*Corresponding Authors:

Bent Smets
[bent.smets@gmail.com](mailto:bent.smets@gmail.com)

Filip Meysman

[filip.meysman@uantwerpen.be](mailto:filip.meysman@uantwerpen.be)

# Supplementary Text

# Supplementary Figures and Tables

## Supplementary Figures


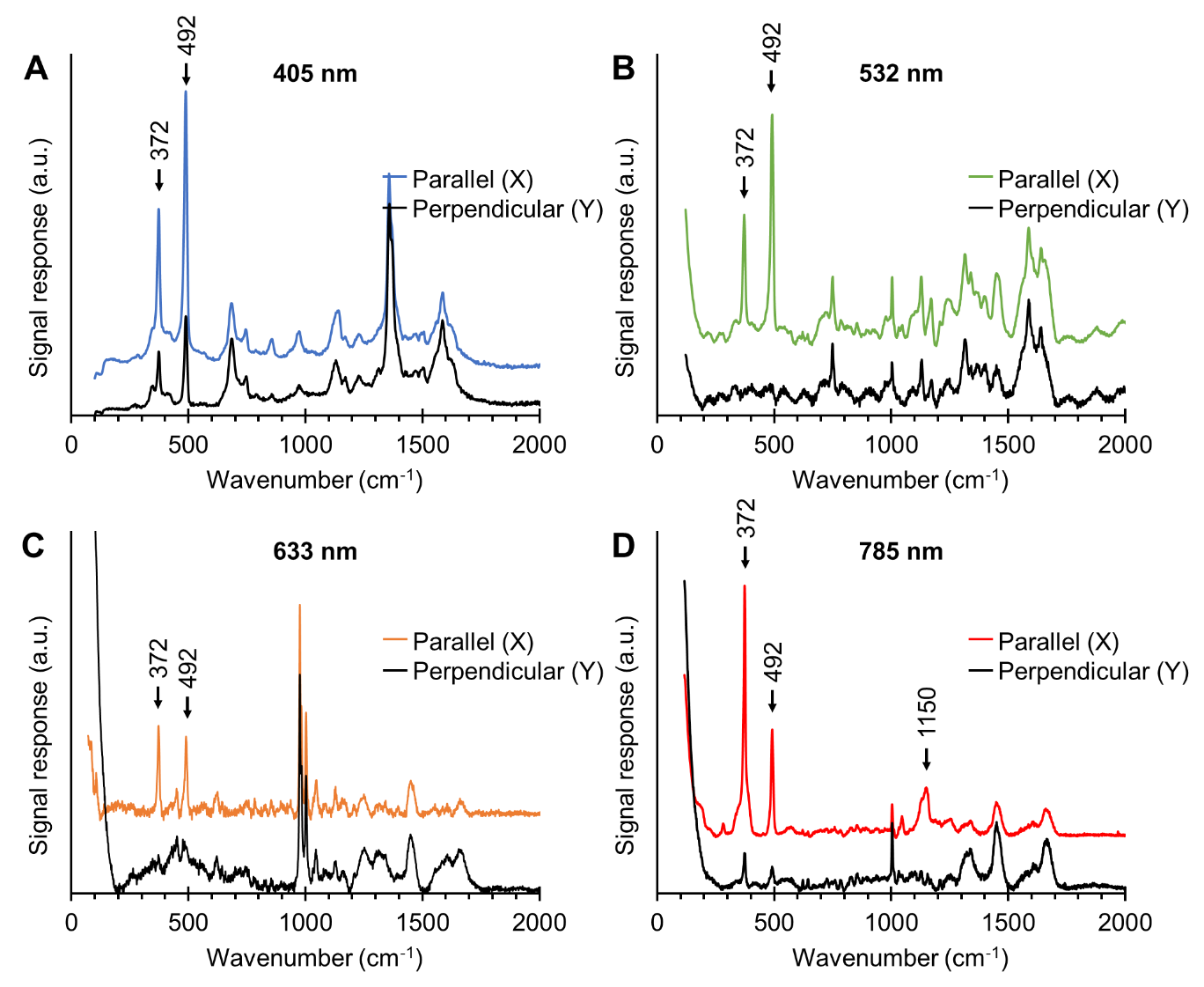


Supplementary Figure 1: Orientation-dependent Raman scattering of the Ni-cofactor in native cable bacterium filaments. The vibrational modes of the Ni-cofactor, indicated with black arrows, show a clear, orientation-dependent response. Raman signals are most intense when fiber sheaths are irradiated with incident laser light (wavelengths are indicated above graphs) that is polarized parallel to the direction of the conductive fibers. Irradiation with perpendicularly polarized light yields a lower Raman signal intensity in cofactor-related modes, while other vibrational modes in cytochromes and proteins remain unaffected. Spectra are off-set for clarity.

## Supplementary Tables

Supplementary Table 1: Complete overview of Raman active vibrational modes found in cable bacteria and fiber sheaths. Dark grey shaded areas indicate modes observed in native cable bacterium filaments (CB) and fiber sheaths (FS). Light grey shaded areas indicate modes attributed to the Ni-cofactor.

| **Peak** | **CB** | **FS** | **Intensity** | | **Annotation** | **Ref** |
| --- | --- | --- | --- | --- | --- | --- |
| 53 cm^-1^ | ? |  | | Low | Ni-cofactor: annotation uncertain | [1] |
| 95 cm^-1^ | ? |  | | Low | Ni-cofactor: annotation uncertain | [1] |
| 121 cm^-1^ | ? |  | | Low | Ni-cofactor: annotation uncertain | [1] |
| 180 cm^-1^ |  |  | | Low | Ni-cofactor: δ(SNiS) – S-Ni-S deformation | [2] |
| 273 cm^-1^ |  |  | | Low | Ni-cofactor: ν(Ni-S)_asym_ – Ni-S stretching | [2] |
| 308 cm^-1^ |  |  | | Low | Ni-cofactor: annotation uncertain | [1] |
| 372 cm^-1^ |  |  | | High | Ni-cofactor: ν(Ni-S)_sym_ – Ni-S stretching | [2,3] |
| 448 cm^-1^ |  |  | | Low | Ni cofactor: annotation uncertain | [1] |
| 492 cm^-1^ |  |  | | High | Ni-cofactor: ν(C-S) + ring deformation | [2,3] |
| 622 cm^-1^ |  |  | | Low | Ni cofactor: annotation uncertain | [1] |
| 680 cm^-1^ |  |  | | Medium | Cytochromes: ν(C_a_-S) | [4,5] |
| 750 cm^-1^ |  |  | | Medium | Cytochromes: ν_15_ – Pyrrole breathing | [4,5] |
| 856 cm^-1^ |  |  | | Low | Tyrosine: Fermi resonance – ν_1_ + 2ν_16a_ | [6] |
| 971 cm^-1^ |  |  | | High | Phosphate: ν(PO_4_^3-^) – Symmetric phosphate stretching | [7,8] |
| 1004 cm^-1^ |  |  | | Medium | Phenyl alanine: Ring breathing | [9] |
| 1048 cm^-1^ |  |  | | Low | Phosphate: ν(PO_4_^3-^) – Symmetric phosphate stretching | [7,8] |
| 1130 cm^-1^ |  |  | | Medium | Cytochromes: ν_22_ – vibrations of side radicals C_b_-CH_3_ | [4,5] |
| 1146 cm^-1^ |  |  | | High | Ni-cofactor: ν(C=S·) – thiocarbonyl radical stretching | [10] |
| 1170 cm^-1^ |  |  | | Medium | Cytochromes: ν_30_ – asymmetric stretching pyrrole half-ring | [4,5] |
| 1181 cm^-1^ |  |  | | High | Ni-cofactor: ν(C=S·) – thiocarbonyl radical stretching | [10] |
| 1219 cm^-1^ |  |  | | High | Ni-cofactor: ν(C=S·) – thiocarbonyl radical stretching | [10] |
| 1230 cm^-1^ |  |  | | Low | Cytochrome c: ν_14_ – C_m_-H in-plane bending | [4,5] |
| 1253 cm^-1^ |  |  | | Low | Proteins: δ(N-H) + ν(C-N) – N-H bending + C-N stretching (Amide III) | [11] |
| 1315 cm^-1^ |  |  | | Low | Cytochrome c: ν_21_ – δ(C_m_H) | [4,5] |
| 1339 cm^-1^ |  |  | | Low | Proteins: complex mode peptide chain (O=C)-C^α^-H | [12] |
| 1340 cm^-1^ |  |  | | Low | Adenine, guanine, tyrosine, tryptophan | [13,14] |
| 1360 cm^-1^ |  |  | | High | Cytochromes: ν_4_ – symmetric pyrrole half-ring stretching | [4,5] |
| 1371 cm^-1^ |  |  | | High | Cytochromes: ν_4_ – symmetric pyrrole half-ring stretching | [4,5] |
| 1399 cm^-1^ |  |  | | Low | Cytochromes: ν20 – stretching of pyrrole quarter-ring | [4,5] |
| 1450 cm^-1^ |  |  | | Medium | Carbohydrates, lipids, protein: ρ(CH_2_) – CH_2_ scissoring | [15-18] |
| 1588 cm^-1^ |  |  | | Medium | Cytochromes: ν_19_ – ν(C_α_C_m_)_asym_ | [4,5] |
| 1640 cm^-1^ |  |  | | Medium | Cytochromes: ν_10_ – ν(C_α_C_m_)_asym_ | [4,5] |
| 1660 cm^-1^ |  |  | | Medium | Proteins: ν(C=O) – Carbonyl stretching (Amide I) | [11] |
| 2330 cm^-1^ |  |  | | Low | Atmospheric N_2_: *v*_1_ – N≡N stretching | [19] |
| 2940 cm^-1^ |  |  | | Low | Carbohydrates, DNA, lipids, proteins: ν(C-H) – C-H stretching | [15,18,20,21] |
| 3063 cm^-1^ |  |  | | Low | DNA, proteins: ν(C-H)_arom_ – Aromatic C-H stretching | [22] |

References: [1] this work [2] Johnson (2004), [3] Boschker et al. (2021), [4] Virdis et al. (2014), [5] Milazzo et al. (2018), [6] Siamwiza et al. (1975), [7] Frost et al. (2016), [8] (Nkebiwe et al. 2022) ,[9] Freire et al. (2017), [10] Petrenko et al. (2006), [11] Miura and Thomas (1995), [12] Tsuboi et al. (2000), [13] Harz et al. (2009), [14] Uzunbajakava et al. (2003), [15] Czamara et al. (2015), [16] Ferraro et al. (2003), [17] Salmaso et al. (1994), [18] Wiercigroch et al. (2017), [19] Petrov et al. (2018), [20] Kengne-Momo et al. (2012), [21] Prescott et al. (1984), [22] Larkin (2011)

Supplementary Table 2: Ratio of the Raman signal intensities of Ni-cofactor modes and Amide I (1660 cm^-1^) obtained with the incident laser light polarized parallel and perpendicular to the direction of the conductive fibers in fiber sheaths.

|  | **Ratio: Peak _Parallel_/Peak _Perpendicular_** | | | | | |
| --- | --- | --- | --- | --- | --- | --- |
| **Laser λ** | **367 cm^-1^** | **496 cm^-1^** | **1146 cm^-1^** | **1181 cm^-1^** | **1219 cm^-1^** | **1660 cm^-1^** |
| 405 nm | 2.2 | 2.3 | / | / | / | / |
| 532 nm | 2.3 | 2.5 | / | / | / | / |
| 633 nm | 3.2 | 3.4 | 2.5 | 2.8 | 2.8 | / |
| 785 nm | 4.6 | 4.3 | 3.1 | 3.7 | 6.3 | 0.9 |

**Supplementary Table 3**: Comparison between the vibrational modes observed in nickel bis(1,2-dithiolene) complexes and the Ni-cofactor in cable bacteria.

| **Mode** | **Ni bis(1,2-dithiolene) complexes** | **Ni-cofactor** |
| --- | --- | --- |
| S-Ni-S deformation | ~180 cm^-1^ | 182 cm^-1^ |
| Asym. Ni-S stretching | ~280 cm^-1^ | 278 cm^-1^ |
| Sym. Ni-S stretching | ~340 cm^-1^ | 367 cm^-1^ |
| C-S stretching + ring deformation | ~500 cm^-1^ | 496 cm^-1^ |
| C=S‧ stretching | 1100 – 1200 cm^-1^ | 1146 cm^-1^, 1181 cm^-1^, 1219 cm^-1^ |

# References

Boschker, Henricus T.S. et al. 2021. “Efficient Long-Range Conduction in Cable Bacteria through Nickel Protein Wires.” *Nature Communications* 12(1): 1–30.

Czamara, K et al. 2015. “Raman Spectroscopy of Lipids: A Review.” *Journal of Raman Spectroscopy* 46(1): 4–20.

Ferraro, John R., Kazuo Nakamoto, and Chris W. Brown. 2003. Introductory Raman Spectroscopy: Second Edition *Introductory Raman Spectroscopy: Second Edition*. Elsevier Inc. (July 28, 2020).

Freire, Paulo T.C. et al. 2017. Raman Spectroscopy and Applications *Raman Spectroscopy of Amino Acid Crystals*. IntechOpen.

Frost, Ray L., Ricardo Scholz, and Andrés López. 2016. “A Raman and Infrared Spectroscopic Study of the Phosphate Mineral Laueite.” *Vibrational Spectroscopy* 82: 31–36. (June 17, 2022).

Harz, M., P. Rösch, and J. Popp. 2009. “Vibrational Spectroscopy-A Powerful Tool for the Rapid Identification of Microbial Cells at the Single-Cell Level.” In *Cytometry Part A*, John Wiley & Sons, Ltd, 104–13.

Johnson, Michael K. 2004. 52 Progress in Inorganic Chemistry *Vibrational Spectra of Dithiolene Complexes*.

Kengne-Momo, R. P. et al. 2012. “Protein Interactions Investigated by the Raman Spectroscopy for Biosensor Applications.” *International Journal of Spectroscopy* 2012(i): 1–7.

Kowalska, Patrycja et al. 2012. “Experimental and Theoretical Polarized Raman Linear Difference Spectroscopy of Small Molecules with a New Alignment Method Using Stretched Polyethylene Film.” *Analytical Chemistry* 84(3): 1394–1401.

Larkin, Peter. 2011. Elsevier *Infrared and Raman Spectroscopy: Principles and Spectral Interpretation*.

Milazzo, Lisa, Lorenzo Tognaccini, Barry D. Howes, and Giulietta Smulevich. 2018. “Probing the Non-Native States of Cytochrome c with Resonance Raman Spectroscopy: A Tool for Investigating the Structure–Function Relationship.” *Journal of Raman Spectroscopy* 49(6): 1041–55.

Miura, Takashi, and George J Thomas. 1995. “Raman Spectroscopy of Proteins and Their Assemblies.” In *Proteins: Structure, Function, and Engineering*, eds. B. B. Biswas and Siddhartha Roy. Boston, MA: Springer US, 55–99.

Nkebiwe, Peteh Mehdi et al. 2022. “Detection of Calcium Phosphate Species in Soil by Confocal μ-Raman Spectroscopy.” *Journal of Plant Nutrition and Soil Science* 185(2): 221–31.

Petrenko, Taras, Kallol Ray, Karl E. Wieghardt, and Frank Neese. 2006. “Vibrational Markers for the Open-Shell Character of Transition Metal Bis-Dithiolenes: An Infrared, Resonance Raman, and Quantum Chemical Study.” *Journal of the American Chemical Society* 128(13): 4422–36.

Petrov, D. V., I. I. Matrosov, D. O. Sedinkin, and A. R. Zaripov. 2018. “Raman Spectra of Nitrogen, Carbon Dioxide, and Hydrogen in a Methane Environment.” *Optics and Spectroscopy (English translation of Optika i Spektroskopiya)* 124(1): 8–12.

Prescott, B., W. Steinmetz, and G. J. Thomas. 1984. “Characterization of DNA Structures by Laser Raman Spectroscopy.” *Biopolymers* 23(2): 235–56.

Salmaso, B. L. et al. 1994. “Resonance Raman Microspectroscopic Characterization of Eosinophil Peroxidase in Human Eosinophilic Granulocytes.” *Biophysical Journal* 67(1): 436–46. (April 4, 2022).

Siamwiza, Mwindaace N. et al. 1975. “Interpretation of the Doublet at 850 and 830 Cm−1 in the Raman Spectra of Tyrosyl Residues in Proteins and Certain Model Compounds.” *Biochemistry* 14(22): 4870–76.

Tsuboi, Masamichi, Mika Suzuki, Stacy A. Overman, and George J. Thomas. 2000. “Intensity of the Polarized Raman Band at 1340-1345 Cm-1 as an Indicator of Protein α-Helix Orientation: Application to Pf1 Filamentous Virus.” *Biochemistry* 39(10): 2677–84.

Uzunbajakava, N. et al. 2003. “Nonresonant Raman Imaging of Protein Distribution in Single Human Cells.” *Biopolymers - Biospectroscopy Section* 72(1): 1–9.

Virdis, Bernardino, Diego Millo, Bogdan C Donose, and Damien J Batstone. 2014. “Real-Time Measurements of the Redox States of c-Type Cytochromes in Electroactive Biofilms : A Confocal Resonance Raman Microscopy Study.” *PLOS* 9(2).

Wiercigroch, Ewelina et al. 2017. “Raman and Infrared Spectroscopy of Carbohydrates: A Review.” *Spectrochimica Acta - Part A: Molecular and Biomolecular Spectroscopy* 185: 317–35.


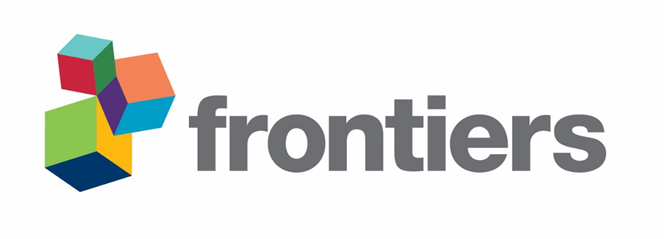

Supplement: Supplementary file 1 [file Data_Sheet_1.docx]
